# Supplementary material for: Properties of winning Iterated Prisoner’s Dilemma strategies
Source: PLoS Comput Biol. 2024 Dec 26;20(12):e1012644. doi: 10.1371/journal.pcbi.1012644 (PMC11670945; doi:10.1371/journal.pcbi.1012644)
Supplement: S1 Text — This document provides details of further analysis, a summary of all parameters and notations used in the manuscript, and a comprehensive list of all strategies considered in this work. (PDF) [file pcbi.1012644.s001.pdf]

Electronic supplementary material
Properties of Winning Iterated Prisoner's Dilemma Strategies

Nikoleta E. Glynatsi, Vincent A. Knight, Marc Harper

This document provides additional details and analysis based on the complete datasets for noisy and probabilistic ending tournaments. In Section A, a summary of all parameters and notation used in the manuscript is presented. Section B offers further details on the correlation analysis and includes additional information. In Section D, we present the results of the correlation analysis when all features of the strategies are considered. In Section E, the results of the random forest and clustering evaluations with the inclusion of all features are discussed. Section F presents the results of the regression analysis on the median score of the strategies. Finally, in Section G, a comprehensive list of all strategies considered in this work is provided.

A Parameters Summary

All the parameters used in this manuscript alongside their explanation are given by Table 1.

Feature	Explanation
SSE	A measure of how far a strategy is from extortionate behaviour defined in [?].
$C_{\max}$	The biggest cooperating rate in the tournament.
$C_{\min}$	The smallest cooperating rate in the tournament.
C_{median}	The median cooperating rate in the tournament.
C_{mean}	The mean cooperating rate in the tournament.
C_r / $C_{\max}$	A strategy's cooperating rate divided by the maximum cooperating rate in the tournament.
$C_{\min}$ / C_r	The minimum in the tournament divided by a strategy's cooperating rate.
C_r / C_{median}	A strategy's cooperating rate divided by the median cooperating rate in the tournament.
C_r / C_{mean}	A strategy's cooperating rate divided by the mean cooperating rate in the tournament.
C_r	The cooperating rate of a strategy.
CC to C rate	The probability a strategy will cooperate after a mutual cooperation.
CD to C rate	The probability a strategy will cooperate after being betrayed by the opponent.
DC to C rate	The probability a strategy will cooperate after betraying the opponent.
DD to C rate	The probability a strategy will cooperate after a mutual defection.
p_n	The probability of a player's action being flipped at each interaction.
n	The number of turns in a match.
p_e	The probability of a match ending in the next turn.
N	The number of strategies in the tournament.
k	The number that a given tournament is repeated.

Table 1: The features which are included in the performance evaluation analysis.

B Top ranked strategies and features analysis for the entire data set

In this section we carry out a similar analysis as the one presented in main manuscript, but this time we use the entire data set for noisy and probabilistic ending tournaments.

B.1 Top ranked strategies across tournaments

The top 15 strategies for each tournament type based on $\bar{r}$ are given in Table 2. The data collection process was designed such that the probabilities of noise and ending of the match varied between 0 and 1. However, commonly used values for these probabilities are values less than 0.1. Thus, Table 2 also includes the top 15 strategies in noisy tournaments with $p_n < 0.1$ and probabilistic ending tournaments with $p_e < 0.1$.

	Standard		Noisy		Noisy ($p_n < 0.1$)		Probabilistic ending		Probabilistic ending ($p_e < 0.1$)		Noisy probabilistic ending	
	Name	$\bar{r}$	Name	$\bar{r}$	Name	$\bar{r}$	Name	$\bar{r}$	Name	$\bar{r}$	Name	$\bar{r}$
0	Evolved HMM 5	0.007	Grumpy	0.14	DBS	0.0	Fortress4	0.013	Evolved FSM 16	0.0	Alternator	0.304
1	Evolved FSM 16	0.01	e	0.19	Evolved FSM 16 Noise 05	0.008	Defector	0.014	Evolved FSM 16 Noise 05	0.013	ϕ	0.31
2	EvolvedLookerUp2_2_2	0.011	Tit For 2 Tats	0.206	Evolved ANN 5 Noise 05	0.013	Better and Better	0.016	MEM2	0.027	e	0.312
3	Evolved FSM 16 Noise 05	0.017	Slow Tit For Two Tats	0.21	BackStabber	0.024	Tricky Defector	0.019	Evolved HMM 5	0.043	π	0.317
4	PSO Gambler 2_2_2	0.022	Cycle Hunter	0.215	DoubleCrosser	0.025	Fortress3	0.022	EvolvedLookerUp2_2_2	0.049	Limited Retaliate	0.353
5	Evolved ANN	0.029	Risky QLearner	0.222	Evolved ANN 5	0.028	Gradual Killer	0.025	Spiteful Tit For Tat	0.059	Anti Tit For Tat	0.354
6	Evolved ANN 5	0.034	Cycler CCCCCD	0.229	Evolved ANN	0.038	Aggravater	0.028	Nice Meta Winner	0.069	Limited Retaliate 3	0.356
7	PSO Gambler 1_1_1	0.037	Retaliate 3	0.23	Spiteful Tit For Tat	0.051	Raider	0.031	NMWE Finite Memory	0.069	Retaliate 3	0.356
8	Evolved FSM 4	0.049	Retaliate 2	0.239	Evolved HMM 5	0.051	Cycler DDC	0.045	NMWE Deterministic	0.07	Retaliate	0.357
9	PSO Gambler Mem1	0.05	Defector Hunter	0.24	Level Punisher	0.052	Hard Prober	0.051	Grudger	0.07	Retaliate 2	0.358
10	Winner12	0.06	Retaliate	0.242	Omega TFT	0.059	SolutionB1	0.06	NMWE Long Memory	0.074	Limited Retaliate 2	0.361
11	Fool Me Once	0.061	Hard Tit For 2 Tats	0.25	Fool Me Once	0.059	Meta Minority	0.061	Nice Meta Winner Ensemble	0.076	Hopeless	0.368
12	DBS	0.071	Arrogant QLearner	0.25	PSO Gambler 2_2_2 Noise 05	0.067	Bully	0.061	EvolvedLookerUp1_1_1	0.077	Arrogant QLearner	0.406
13	DoubleCrosser	0.072	Limited Retaliate 3	0.253	Evolved FSM 16	0.078	EasyGo	0.071	NMWE Memory One	0.08	Cautious QLearner	0.409
14	BackStabber	0.075	ShortMem	0.253	EugineNier	0.08	Fool Me Forever	0.071	NMWE Stochastic	0.085	Fool Me Forever	0.418

Table 2: Top performances for each tournament type based on $\bar{r}$. The results of each type are based on 11420 unique tournaments. The results for noisy tournaments with $p_n < 0.1$ are based on 1151 tournaments, and for probabilistic ending tournaments with $p_e < 0.1$ on 1139. The top ranks indicate that trained strategies perform well in a variety of environments, but so do simple deterministic strategies. The normalised medians are close to 0 for most environments, except environments with noise not restricted to 0.1 regardless of the number of turns. Noisy and noisy probabilistic ending tournaments have the highest medians.

The r distributions for the top ranked strategies of Table 2 are given by Figure 1.

In standard tournaments 10 out of the 15 top strategies were introduced in [22]. These are strategies based on finite state automata (FSM), hidden Markov models (HMM), artificial neural networks (ANN), lookup tables (LookerUp) and stochastic lookup tables (Gambler) that have been trained using reinforcement learning algorithms (evolutionary and particle swarm algorithms). They have been trained to perform well against a subset of the strategies in APL in a standard tournament, thus their performance in the specific setting was anticipated although still noteworthy given the random sampling of tournament participants. DoubleCrosser, BackStabber and Fool Me Once, are strategies not from the literature but from the APL. DoubleCrosser is an extension of BackStabber and both strategies make use of the number of turns because they are set to defect on the last two rounds. It should be noted that these strategies can be characterised as “cheaters” because the source code of the strategies allows them to know the number of turns in a match (unless the match has a probabilistic ending). These strategies were expected to not perform as well in tournaments where the number of turns is not specified. Finally, Winner 12 [31] and DBS [9] are both from the literature. DBS is a strategy specifically designed for noisy environments, however, it ranks highly in standard tournaments as well. Similarly the fourth ranked player, Evolved FSM 16 Noise 05, was trained for noisy tournaments yet performs well in standard tournaments. Figure 1a shows that these strategies typically perform well in any standard tournament in which they participate.

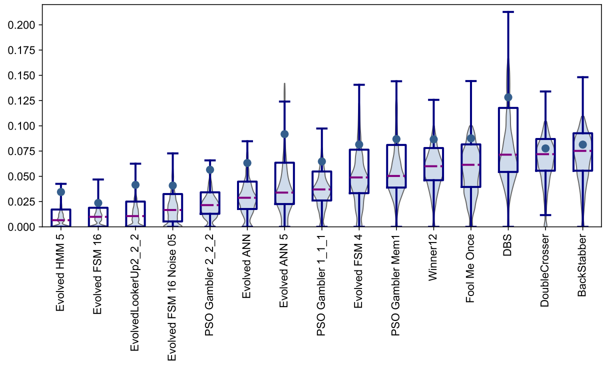

(a) r distributions of top 15 strategies in standard tournaments.

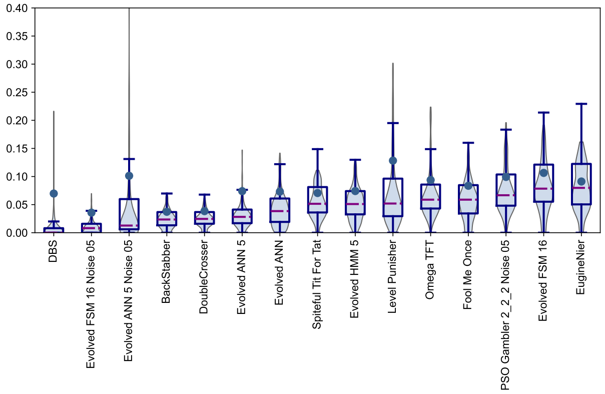

(b) r distributions of top 15 strategies in noisy tournaments with $p_n < 0.1$.

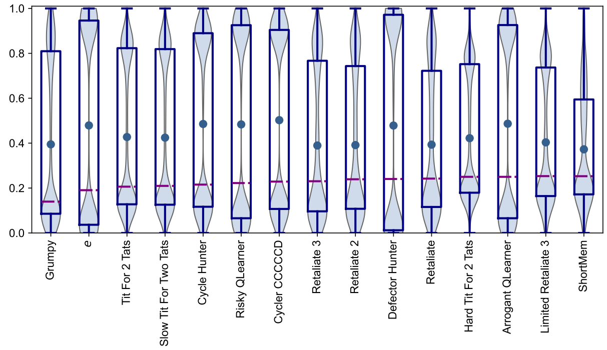

(c) r distributions of top 15 strategies in noisy tournaments.

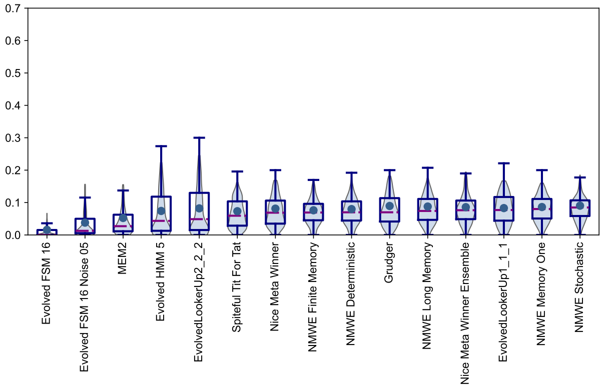

(d) r distributions of top 15 strategies in 1139 probabilistic ending tournaments with $p_e < 0.1$.

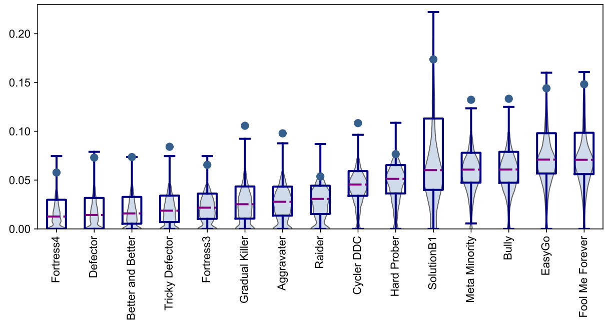

(e) r distributions of top 15 strategies in probabilistic ending tournaments.

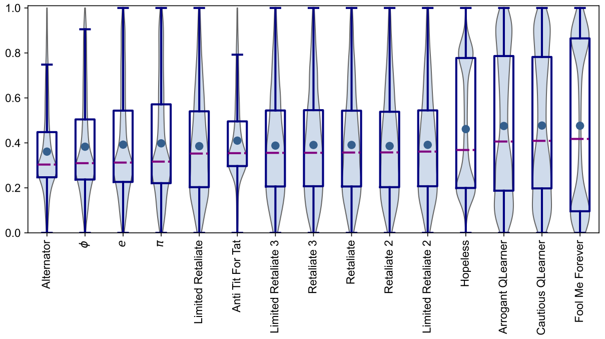

(f) r distributions of top 15 strategies in noisy probabilistic ending tournaments.

Figure 1: r distributions of the top 15 strategies in different environments. A lower value of $\bar{r}$ corresponds to a more successful performance. A strategy's r distribution skewed towards zero indicates that the strategy ranked highly in most tournaments it participated in. Most distributions are skewed towards zero except the distributions with unrestricted noise, supporting the conclusions from Table 2.

In the case of noisy tournaments with smaller noise $p_n < 0.1$ the top performing strategies include strategies specifically designed for noisy tournaments. These are DBS, Evolved FSM 16 Noise 05, Evolved ANN 5 Noise 05, PSO Gambler 2 2 2 Noise 05 and Omega Tit For Tat [25]. Omega TFT, a strategy designed to break the deadlocking cycles of CD and DC that TFT can fall into in noisy environments, places 10th. The rest of the top ranks are occupied by strategies which performed well in standard tournaments and deterministic strategies such as Spiteful Tit For Tat [1], Level Punisher [18], Eugine Nier [35].

In contrast, the performance of the top ranked strategies in noisy environments when $p_n \in [0, 1]$ is bimodal. The top strategies include strategies which decide their actions based on the cooperation to defection ratio, such as ShortMem [17], Grumpy [40] and e [40], and the Retaliate strategies which are designed to defect if the opponent has tricked them more often than a given percentage of the times that they have done the same. The bimodality of the r distributions is explained by Figure 2 which demonstrates that the top 6 strategies were highly ranked due to the their performance in tournaments with $p_n > 0.5$, and that in tournaments with $p_n < 0.5$ they performed poorly. At a noisy level of 0.5 or greater, mostly cooperative strategies become mostly defectors and vice versa.

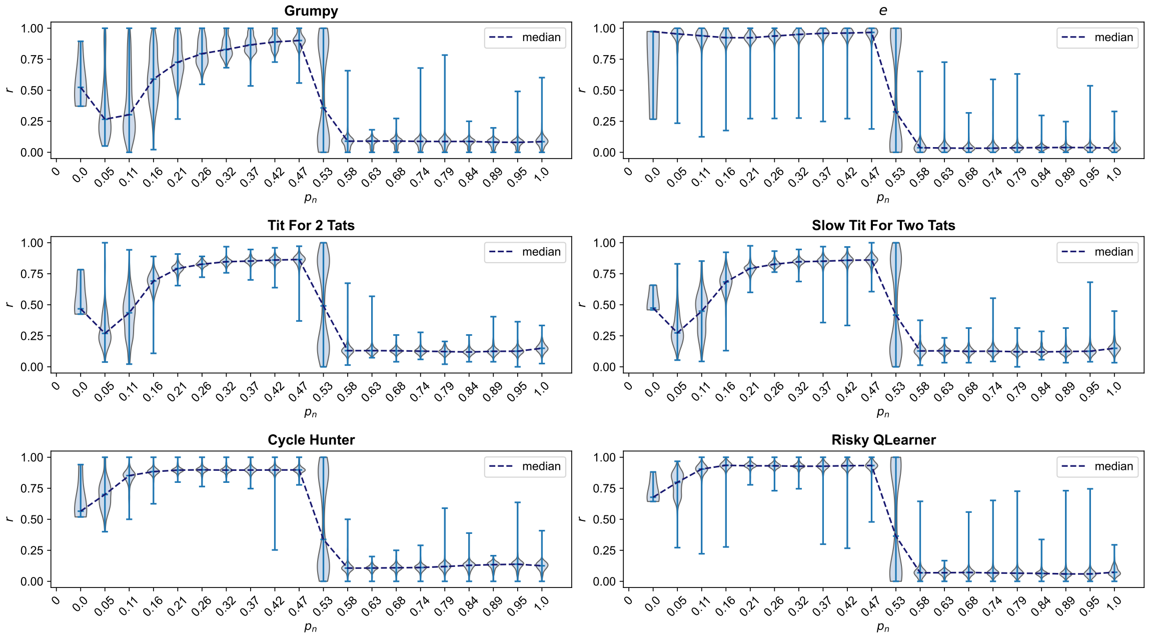

Figure 2: Normalised rank r distributions for top 6 strategies in noisy tournaments over the probability of noisy (p_n).

The most effective strategies in probabilistic ending tournaments with $p_e < 0.1$ are a series of ensemble Meta strategies, trained strategies which performed well in standard tournaments, and Grudger [40] and Spiteful Tit for Tat [1]. The Meta strategies [40] utilize a team of strategies and aggregate the potential actions of the team members into a single action in various ways. Figure 1d indicates that these strategies performed well in any probabilistic ending tournament.

In probabilistic ending tournaments with $p_e \in [0, 1]$ the top ranks are mostly occupied by defecting strategies such as Better and Better, Gradual Killer, Hard Prober (all from [40]), Bully (Reverse Tit For Tat) [33] and Defector, and a series of strategies based on finite state automata introduced by Daniel Ashlock and Wendy Ashlock: Fortress 3, Fortress 4 (both introduced in [7]), Raider [8] and Solution B1 [8]. The success of defecting strategies in probabilistic ending tournaments is due to larger values of p_e which lead to shorter matches (the expected number of rounds is $1/p_e$), so the impact of the PD being iterated is subdued. This is

captured by the Folk Theorem [20] as defecting strategies do better when the likelihood of the game ending in the next turn increases. This is demonstrated by Figure 3, which gives the distributions of r for the top 6 strategies in probabilistic ending tournaments over p_e.

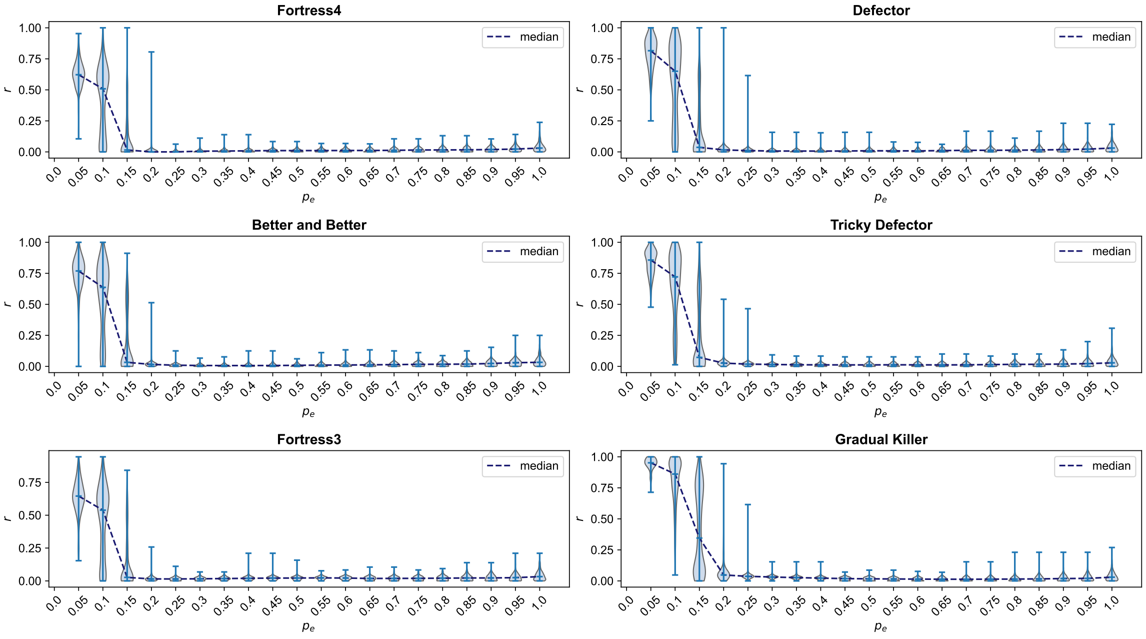

Figure 3: Normalised rank r distributions for top 6 strategies in probabilistic ending tournaments over p_e. The 6 strategies start of with a high median rank, however, their ranked decreased as the the probability of the game ending increased and at the point of $p_e = 0.1$.

The top performances in tournaments with both noise and a probabilistic ending have the largest median values compared to the top rank strategies of the other tournament types. The $\bar{r}$ for the top strategy is approximately at 0.3, indicating that the most successful strategy can on average just place in the top 30% of the competition.

On the whole, the analysis has shown that:

- In standard tournaments the dominating strategies were strategies that had been trained using reinforcement learning techniques.
- In noisy environments where the noise probability strictly less than 0.1 was considered, the successful strategies were strategies specifically designed or trained for noisy environments.
- In probabilistic ending tournaments most of the highly ranked strategies were defecting strategies and trained finite state automata, all by the authors of [7, 8]. These strategies ranked high due to their performance in tournaments where the probability of the game ending after each turn was bigger than 0.1.
- In probabilistic tournaments with p_e less than 0.1 the highly ranked strategies were strategies based on the behaviour of others.
- From the collection of strategies considered here, no strategy can be consistently successful in noisy environments, except if the value of noise is constrained to less than a 0.1.

C The effect of strategy features on performance

The correlation coefficients between the strategies' features and the median score and the median normalised rank for the full dataset of tournaments are given by Table 3. The correlation coefficients between all features have been calculated and a graphical representation can be found in the Section D.

	Standard		Noisy		Probabilistic ending		Noisy probabilistic ending	
	r	median score	r	median score	r	median score	r	median score
CC to C rate	-0.501	0.501	0.413	-0.504	0.408	-0.323	0.260	0.023
CD to C rate	0.226	-0.199	0.457	-0.331	0.320	-0.017	0.205	-0.220
DC to C rate	0.127	-0.100	0.509	-0.504	-0.018	0.033	0.341	-0.016
DD to C rate	0.412	-0.396	0.533	-0.436	-0.103	0.176	0.378	-0.263
C_r	-0.323	0.383	0.711	-0.678	0.714	-0.832	0.579	-0.136
C_{max}	0.000	0.050	0.000	0.023	-0.000	0.046	0.000	-0.004
C_{min}	0.000	0.085	-0.000	-0.017	-0.000	0.007	-0.000	0.041
C_{median}	0.000	0.209	-0.000	0.240	0.000	0.187	0.000	0.673
C_{mean}	0.000	0.229	-0.000	0.271	0.000	0.200	-0.000	0.690
C_r / C_{max}	-0.323	0.381	0.616	-0.551	0.715	-0.833	0.536	-0.117
C_{min} / C_r	0.109	-0.080	-0.358	0.250	-0.134	0.151	-0.368	0.113
C_r / C_{median}	-0.330	0.353	0.652	-0.669	0.713	-0.852	0.330	-0.466
C_r / C_{mean}	-0.331	0.357	0.731	-0.740	0.721	-0.861	0.650	-0.621
N	-0.000	-0.009	0.000	0.002	0.000	0.003	0.000	0.001
k	-0.000	-0.002	0.000	0.002	0.000	0.001	0.000	-0.008
n	-0.000	-0.125	-0.000	-0.024	-	-	-	-
p_n	-	-	0.000	0.207	-	-	0.000	-0.650
p_e	-	-	-	-	0.000	0.165	0.000	-0.058
Make use of game	-0.003	-0.022	0.025	-0.082	-0.053	-0.108	0.013	-0.016
Make use of length	-0.158	0.124	0.005	-0.123	-0.025	-0.090	0.014	-0.016
SSE	0.473	-0.452	0.463	-0.337	-0.157	0.224	0.305	-0.259
stochastic	0.006	-0.024	0.022	-0.026	0.002	-0.130	0.021	-0.013
memory usage	-0.098	0.108	-0.009	-0.017	-	-	-	-

Table 3: Correlations between the strategies' features and the normalised rank and the median score.

In standard tournaments the features CC to C, C_r, C_r/C_{max} and the cooperating ratio compared to C_{median} and C_{mean} have a moderately negative effect on the normalised rank (smaller rank is better), and a moderate positive on the median score. The SSE error and the DD to C rate have the opposite effects. Thus, in standard tournaments behaving cooperatively corresponds to a more successful performance. Even though being nice generally pays off that does not hold against defective strategies. Being more cooperative after a mutual defection, that is not retaliating, is associated to lesser overall success in terms of normalised rank. Figure 4 confirms that the winners of standard tournaments always cooperate after a mutual cooperation and almost always defect after a mutual defection.

Compared to standard tournaments, in both noisy and in probabilistic ending tournaments the higher the rates of cooperation the lower a strategy's success and median score. A strategy would want to cooperate less than both the mean and median cooperator in such settings. In probabilistic ending tournaments the correlation coefficients have larger values, indicating a stronger effect. Thus a strategy will be punished more by its cooperative behaviour in probabilistic ending environments, supporting the results of the previous subsection as well. The distributions of the C_r of the winners in both tournaments are given by Figure 5. It confirms that the winners in noisy tournaments cooperated less than 35% of the time and in probabilistic ending tournaments less than 10%. In noisy probabilistic ending tournaments and over all the tournaments' results, the only features that had a moderate effect are $C_r/C_{\text{mean}}, C_r/C_{\text{max}}$ and C_r. In such environments cooperative behaviour appears to be punished less than in noisy and probabilistic ending tournaments.

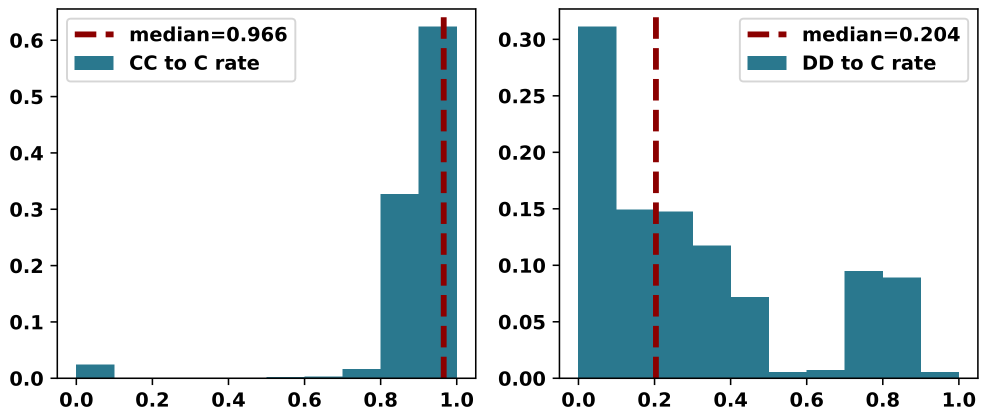

Figure 4: Distributions of CC to C and DD to C for the winners in standard tournaments.

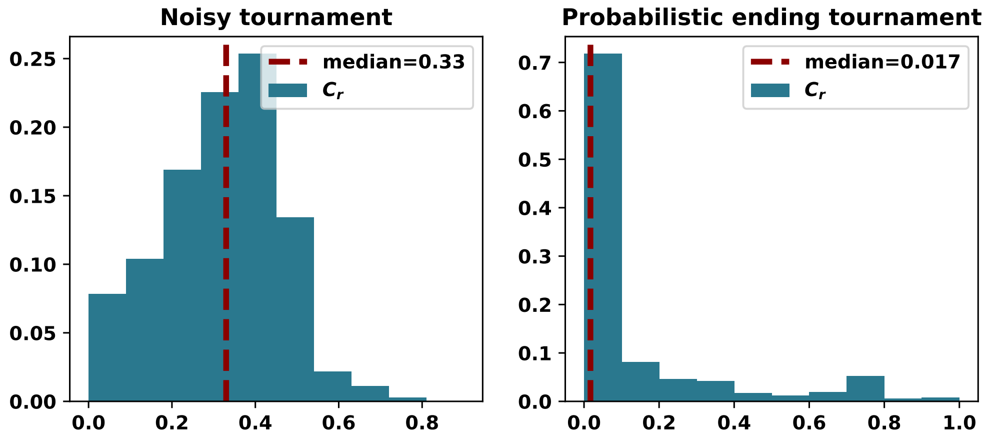

Figure 5: C_r distributions of the winners in noisy and in probabilistic ending tournaments.

A multivariate linear regression has been fitted to model the relationship between the features and the normalised rank. Based on the graphical representation of the correlation matrices given in Section D several of the features are highly correlated and have been removed before fitting the linear regression model. The features included are given by Table 4 alongside their corresponding p values in the distinct tournaments and their regression coefficients.

	Standard		Noisy		Probabilistic ending		Noisy probabilistic ending	
	R adjusted: 0.541		R adjusted: 0.588		R adjusted: 0.587		R adjusted: 0.471	
	Coefficient	p-value	Coefficient	p-value	Coefficient	p-value	Coefficient	p-value
constant	0.695	0.000	0.443	0.0	-0.057	0.018	0.004	0.031
CC to C rate	-0.042	0.000	0.150	0.0	0.017	0.000	0.197	0.000
CD to C rate	0.297	0.000	-0.034	0.0	0.182	0.000	0.022	0.000
DC to C rate	0.198	0.000	0.064	0.0	-0.030	0.000	0.090	0.000
SSE	0.258	0.000	0.237	0.0	-0.041	0.000	0.144	0.000
C_{max}	-0.068	0.000	-	-	-0.021	0.403	-0.090	0.000
C_{min}	-0.161	0.000	1.068	0.0	-0.170	0.000	-	-
C_{mean}	0.117	0.000	-0.722	0.0	-0.024	0.000	-0.112	0.000
C_{min} / C_r	0.057	0.000	-0.544	0.0	0.125	0.000	-	-
C_r / C_{mean}	-0.468	0.000	0.272	0.0	0.525	0.000	0.403	0.000
k	0.000	0.325	0.000	0.1	0.000	0.002	0.001	0.000
n	0.000	0.000	-	-	-	-	-	-
memory usage	-0.010	0.000	0.002	0.0	-	-	-	-
p_n	-	-	-0.039	0.0	-	-	-	-
p_e	-	-	-	-	0.000	0.757	-0.149	0.000

Table 4: Results of multivariate linear regressions with r as the dependent variable. R squared is reported for each model.

A multivariate linear regression has also be fitted on the median score. The coefficients and p values of the features can be found in Section F. This approach leads to similar conclusions.

The feature C_r/C_{mean} has a statistically significant effect across all models and a high regression coefficient. It has both a positive and negative impact on the normalised rank depending on the environment. For standard tournaments, Figure 6 gives the distributions of several features for the winners of standard tournaments. The C_r/C_{mean} distribution of the winner is also given in Figure 6. A value of $C_r/C_{\text{mean}} = 1$ implies that the cooperating ratio of the winner was the same as the mean cooperating ratio of the tournament, and in standard tournaments, the median is 1. Therefore, an effective strategy in standard tournaments was the mean cooperator of its respective tournament.

The distributions of SSE and CD to C rate for the winners of standard tournaments are also given in Figure 6. The SSE distributions for the winners indicate that the strategy behaved in a ZD way in several tournaments, however, not constantly. The winners participated in matches where they did not try to extortionate their opponents. Furthermore, the CD to C distribution indicates that if a strategy were to defect against the winners the winners would reciprocate on average with a probability of 0.5.

Similarly for the rest of the different tournaments types, and the entire data set the distributions of C_r/C_{mean}, SSE and CD to C ratio are given by Figures 7, 9, 10 and 11.

Based on the C_r/C_{mean} distributions the successful strategies have adapted differently to the mean cooperator depending on the tournament type. In noisy tournaments where the median of the distribution is at 0.67,

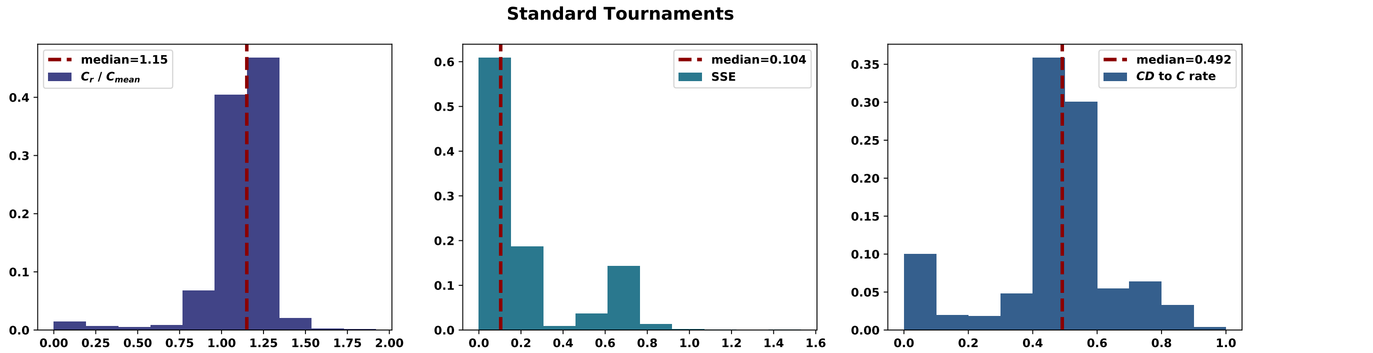

Figure 6: Distributions of C_r/C_{mean}, SSE and CD to C ratio for the winners of standard tournaments. A value of $C_r/C_{\text{mean}} = 1$ imply that the cooperating ratio of the winner was the same as the mean cooperating ratio of the tournament. An SSE distribution skewed towards 0 indicates a extortionate behaviour by the strategy.

and thereupon the winners cooperated 67% of the time the mean cooperator did. In tournaments with noise and a probabilistic ending the winners cooperated 60%, whereas in settings that the type of the tournament can vary between all the types the winners cooperated 67% of the time the mean cooperator did. Lastly, in probabilistic ending tournaments above more defecting strategies prevail (Section **??**), and this result is reflected here.

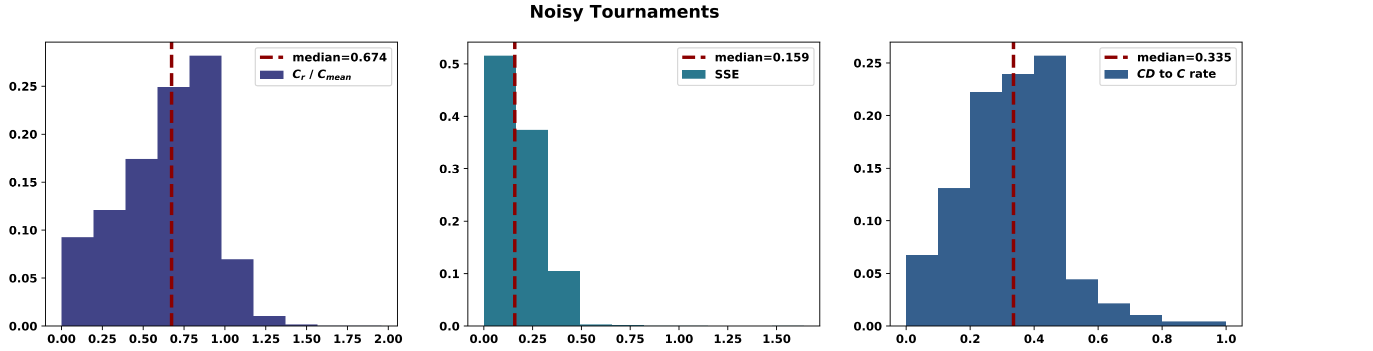

Figure 7: Distributions of C_r/C_{mean}, SSE and CD to C ratio for the winners of noisy tournaments.

The probability of noise has been observed to substantially affect optimal behaviour. Figure 8 gives the ratio C_r/C_{mean} for the winners in tournaments with noise, over the probability of noise. From Figure 8a it is clear that the cooperating only 67% of the time the mean cooperator did is optimal only when $p_n \in [0.2, 0.4)$ and $p_n \in [0.6, 0.7]$. In environments with $p_n < 0.1$ the winners want to be close to the mean cooperator, similarly to standard tournaments, and as the probability of noise is exceeding 0.5 (where the game is effectively inverted) strategies should aim to be less and less cooperative.

Figure 8 gives C_r/C_{mean} for the winners over p_n in tournaments with noise and a probabilistic ending. The optimal proportions of cooperations are different now that the number of turns is not fixed, successful strategies want to be more defecting that the mean cooperator, that only changes when p_n approaches 0.5. Figure 8 demonstrates how the adjustments to C_r/C_{mean} change over the noise in the to the environment, and thus supports how important adapting to the environment is for a strategy to be successful.

The distributions of the SSE across the tournament types suggest that successful strategies exhibit some

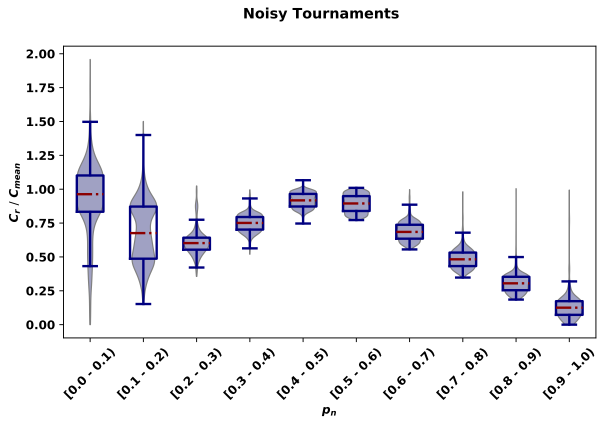

(a) C_r/C_{mean} distribution for winners in noisy tournaments over p_n.

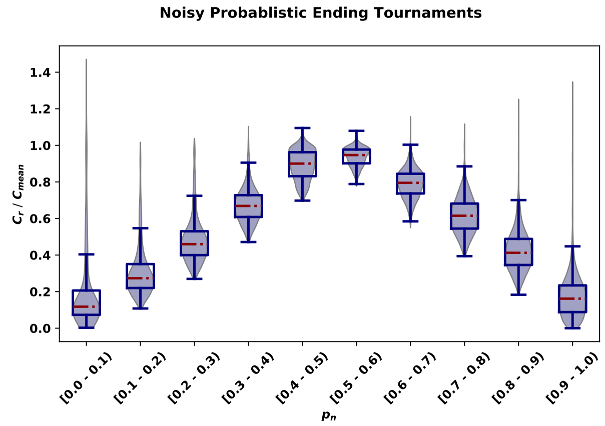

(b) C_r/C_{mean} distribution for winners in noisy probabilistic ending tournaments over p_n.

Figure 8: C_r/C_{mean} distributions over intervals of p_n. These distributions model the optimal proportion of cooperation compared to C_{mean} as a function of (p_n).

extortionate behaviour, but not constantly. ZDs are a set of strategies that are often envious as they try to exploit their opponents. The winners of the tournaments considered in this work are envious, but not as much as many ZDs.

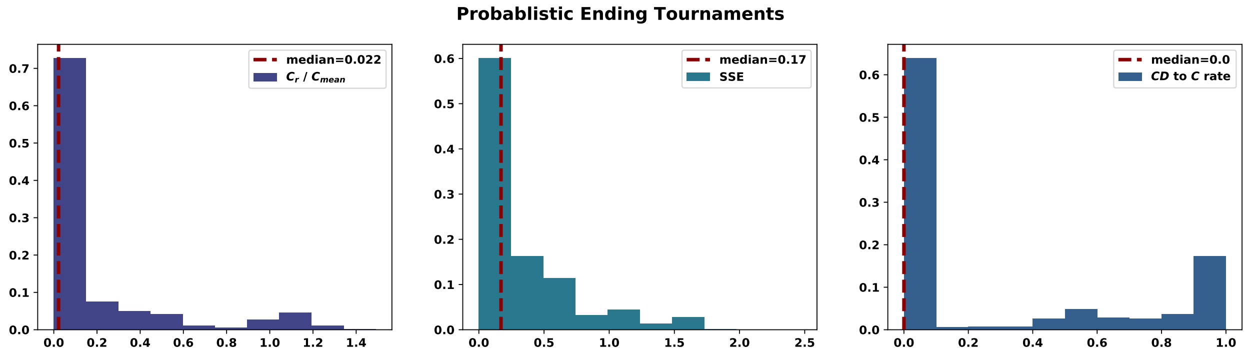

Figure 9: Distributions of C_r/C_{mean}, SSE and CD to C ratio for the winners of probabilistic ending tournaments.

The distributions of the CD to C rate evaluate the behaviour of a successful strategy after its opponent has defected against it. In standard tournaments it was observed that a successful strategy reciprocates with a probability of 0.5, and in a setting that the type of the tournament can vary between all the examined types a winning strategy would reciprocate on average with a probability of 0.58. In tournaments with noise a strategy is less likely to cooperate following a defection compared to standard tournaments, and in probabilistic ending tournaments a strategy will reciprocate a defection. This leads to adjusting the recommendation of being provocable to defections made by Axerlod. A strategy should be provocable in tournaments with short matches, but in the rest of the settings a strategy should be more generous.

Further statistically significant features with strong effects include C_r/C_{min}, C_r/C_{max}, C_{min} and C_{max}. These add more emphasis on how important it is for a a strategy to adapt to its environment. Finally, the features number of turns, repetitions and the probabilities of noise and the game ending had no significant effects based on the multivariate regression models.

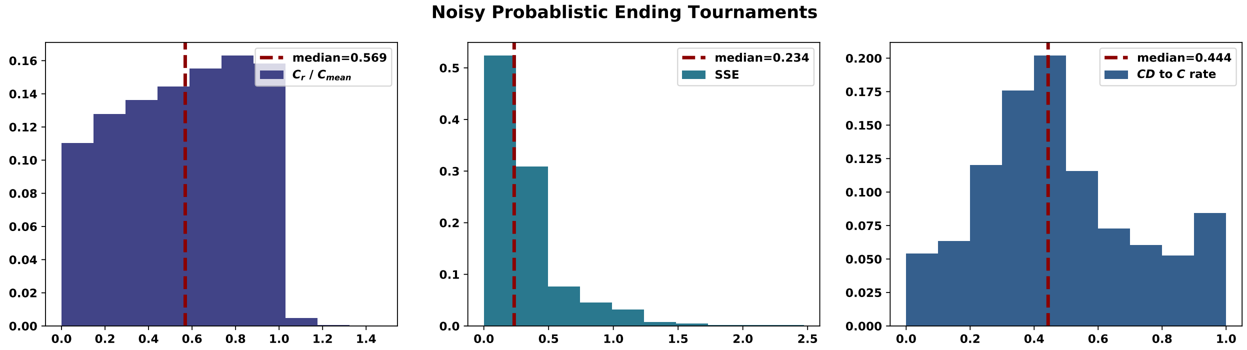

Figure 10: Distributions of C_r/C_{mean}, SSE and CD to C ratio for the winners of noisy probabilistic ending tournaments.

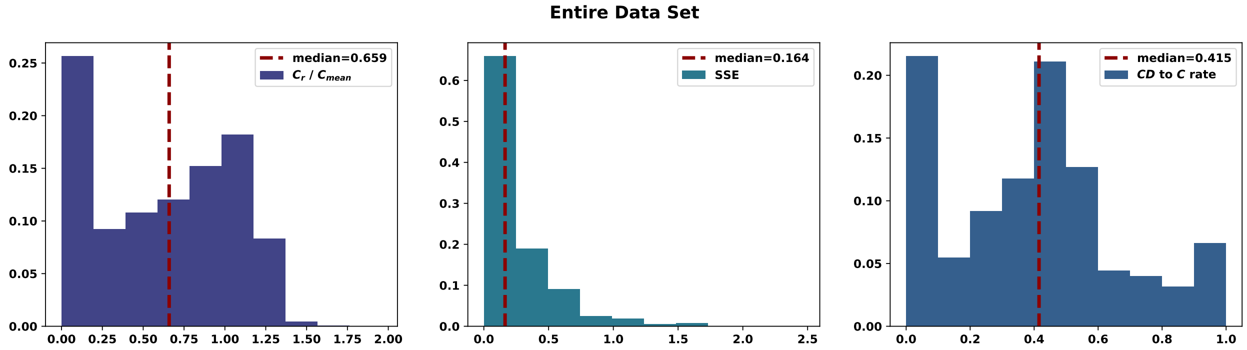

Figure 11: Distributions of C_r/C_{mean}, SSE and CD to C ratio for the winners over the tournaments of the entire data set.

A third method that evaluates the importance of features using clustering and random forests can be found in the Section E. The results uphold the outcomes of the correlation and multivariate regression. It also evaluates the effects of the whether or not a strategy is stochastic, makes use of the knowledge of the utility values, or makes use of match length. These were not evaluated by the methods above because there are binary variables. The results showed that they have no significant effect on a strategy's performance.

D Correlation coefficients

In this section we present the correlation coefficients for the features presented in Table 3 of the main manuscript. The correlation coefficients are calculated using the Spearman's rank correlation coefficient. The results are presented as a graphical representation.

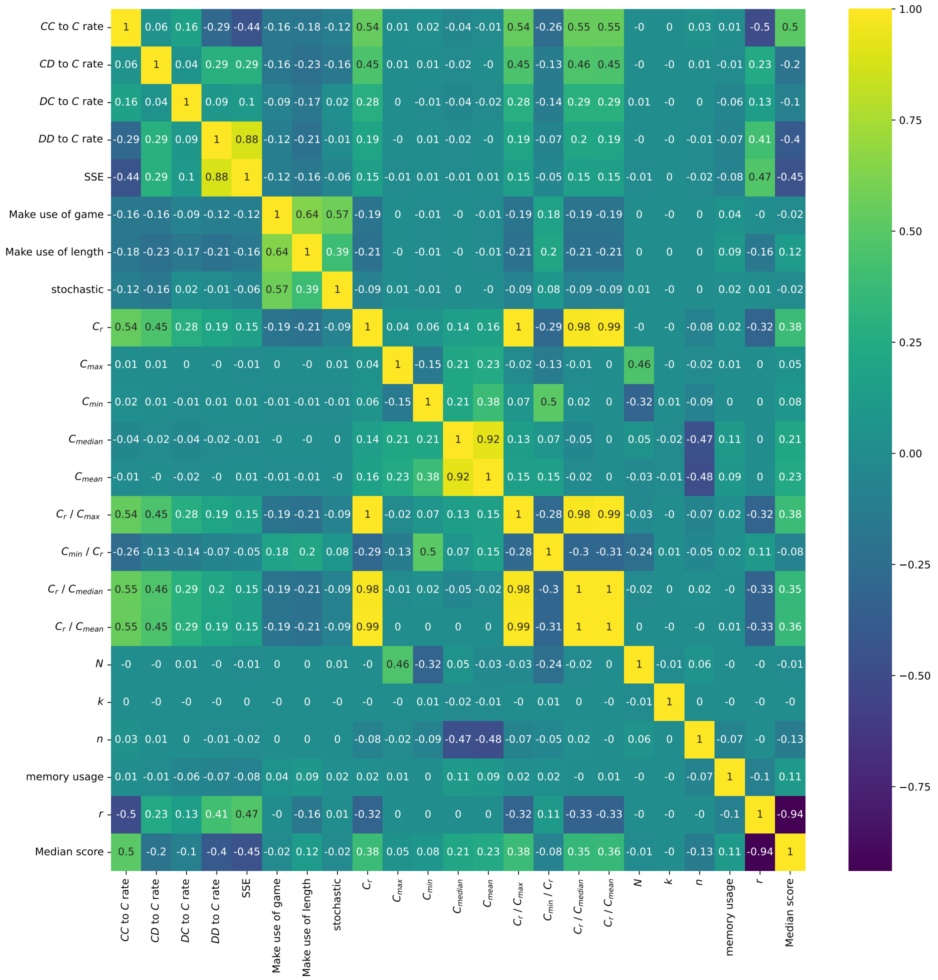

Figure 12: Correlation coefficients of strategies' features for standard tournaments.

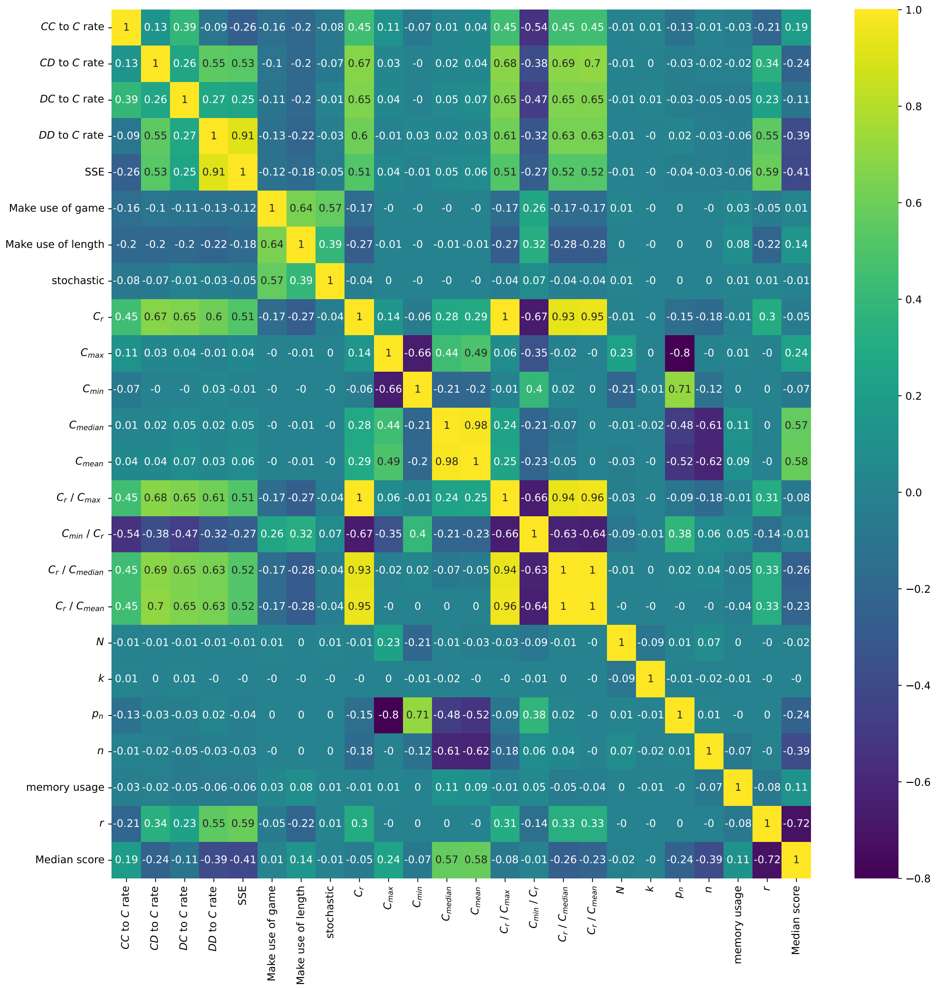

Figure 13: Correlation coefficients of strategies' features for noisy tournaments with $p_n \leq 0.1$.

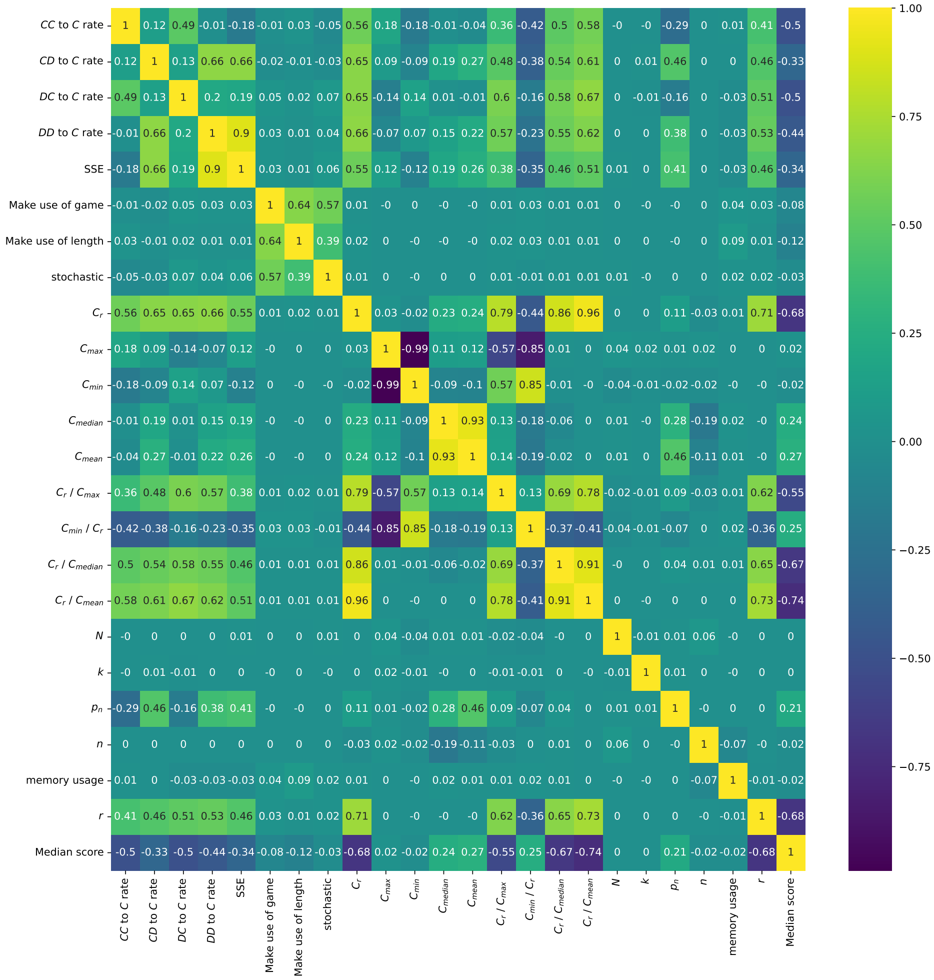

Figure 14: Correlation coefficients of strategies' features for noisy tournaments.

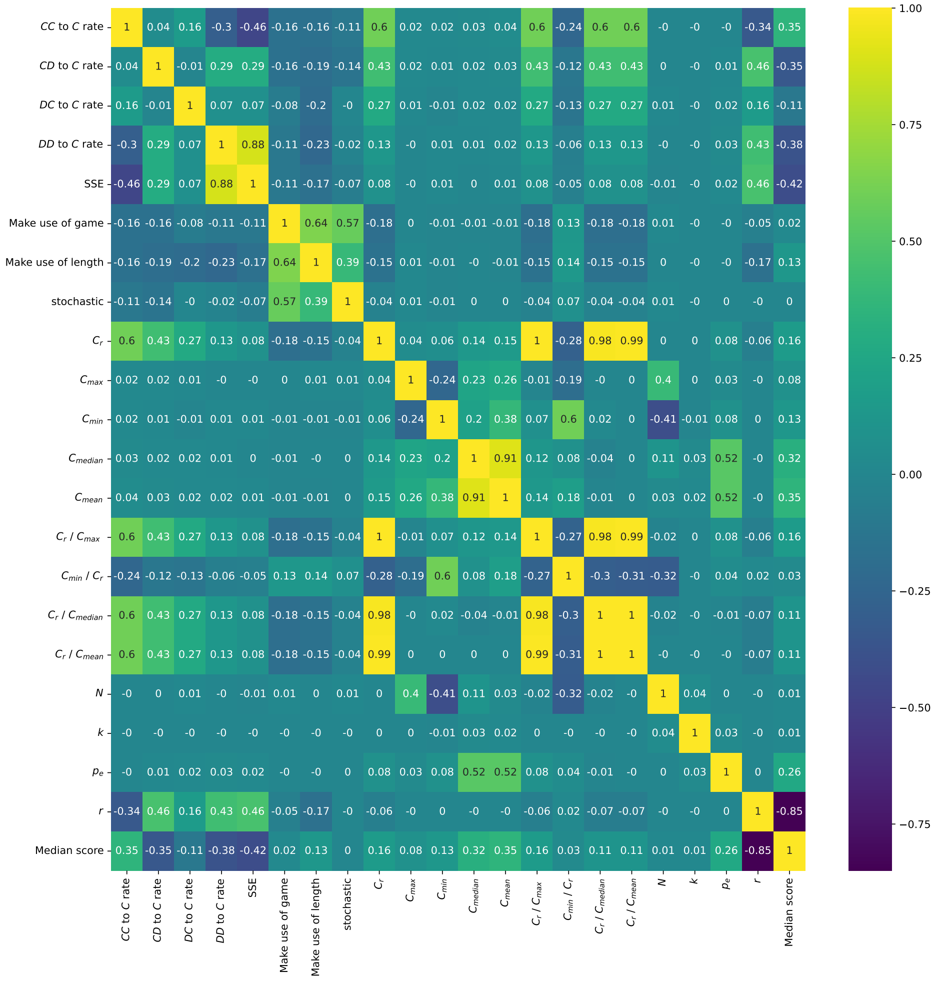

Figure 15: Correlation coefficients of strategies' features for probabilistic ending tournaments with $p_e \leq 0.1$.

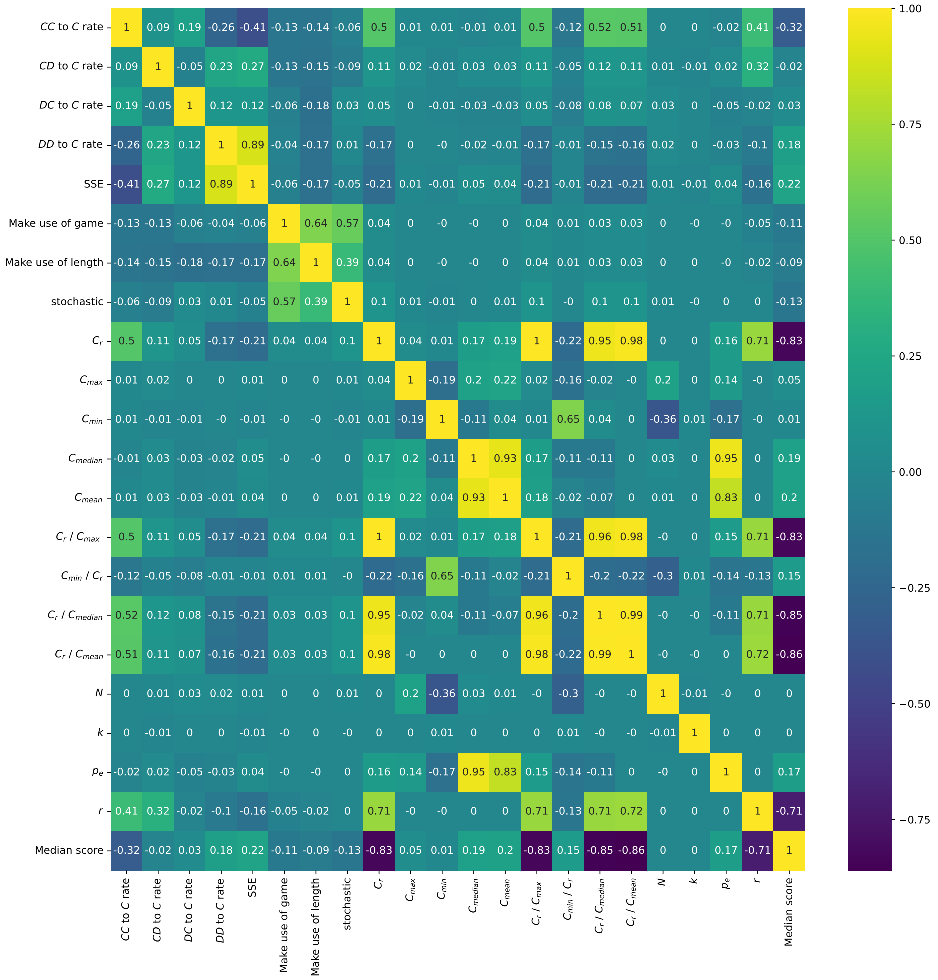

Figure 16: Correlation coefficients of strategies' features for probabilistic ending tournaments.

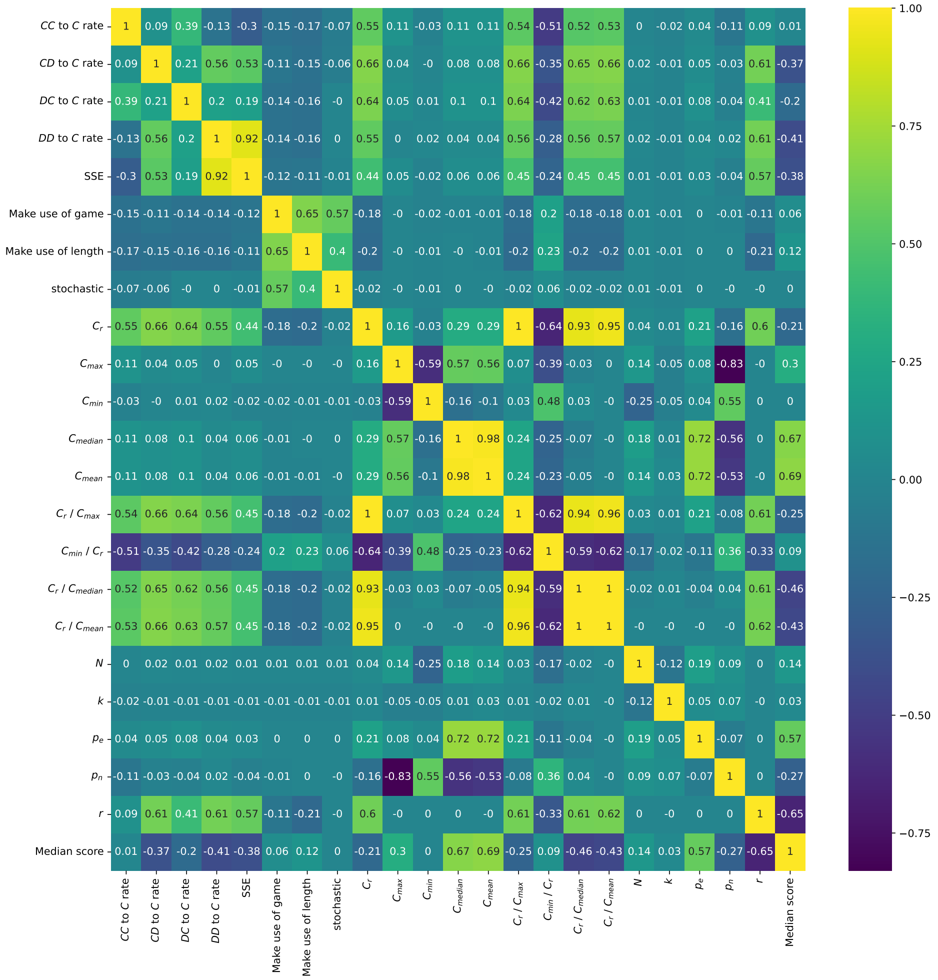

Figure 17: Correlation coefficients of strategies' features for noisy probabilistic ending tournaments with $p_n \leq 0.1$ and $p_e \leq 0.1$.

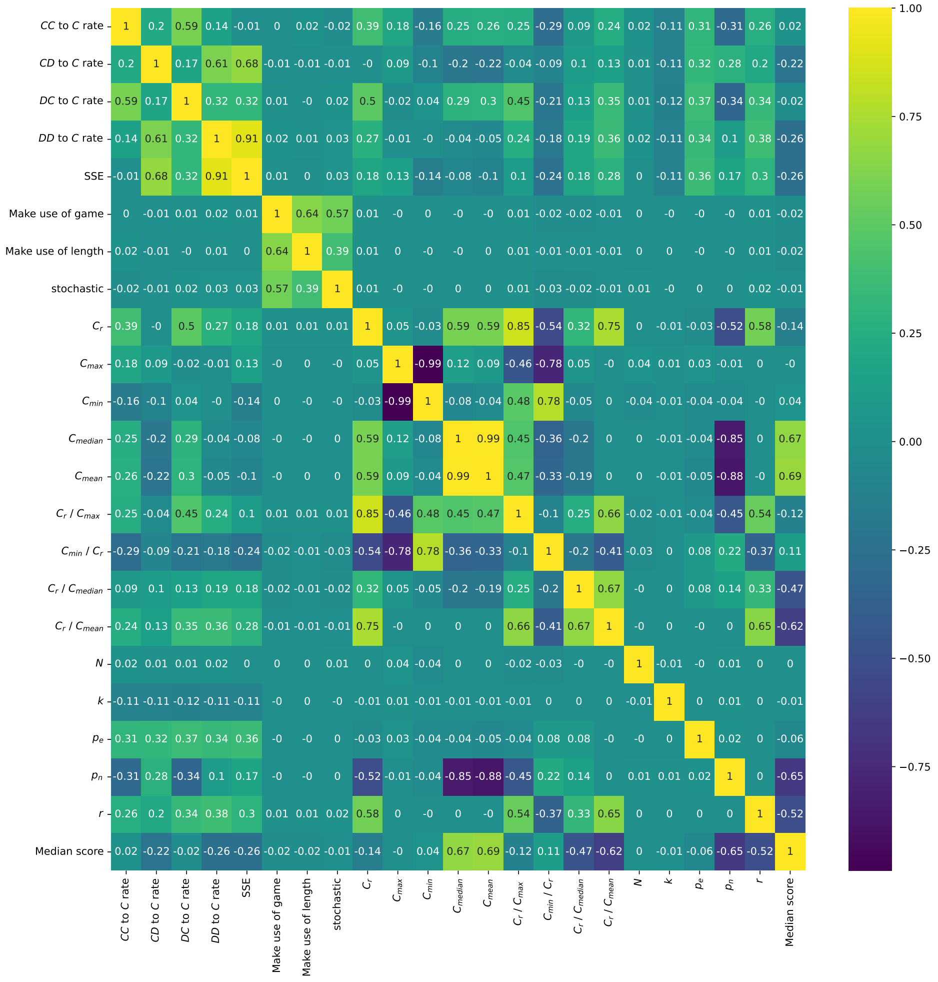

Figure 18: Correlation coefficients of strategies' features for noisy probabilistic ending tournaments.

E Evaluation based on clustering and random forest.

The final method to evaluate the features importance in a strategy's success is a combination of a clustering task and a random forest algorithm. Initially the performances are clustered into different clusters based on them being successful or not. The performances are clustered into successful and unsuccessful clusters based on 4 different approaches. More specifically:

- **Approach 1:** The performances are divided into two clusters based on whether their performance was in the top 5% of their respective tournaments. Thus, whether r was smaller or larger than 0.05.
- **Approach 2:** The performances are divided into two clusters based on whether their performance was in the top 25% of their respective tournaments. Thus, whether r was smaller or larger than 0.25.
- **Approach 3:** The performances are divided into two clusters based on whether their performance was in the top 50% of their respective tournaments. Thus, whether r was smaller or larger than 0.50.
- **Approach 4:** The performances are clustered based on their normalised rank and their median score by a $k-$means algorithm [4]. The number of clusters is not deterministically chosen but it is based on the silhouette coefficients [38].

Once the performances have been assigned to a cluster for each approach a random forest algorithm [15] is applied. The problem is a supervised problem where the random forest algorithm predicts the cluster to which a performance has been assigned to using the features of Table **??**. The random forest models are trained on a training set of 70% of the tournaments results. The accuracy of each model based on R^2 and the number of clusters for each tournament type (because in the case of Approach 4 it is not deterministically chosen) are given by Table 5. The out of the bag error (OOB) [23] has also been calculated. The models fit well, and a high value of both the accuracy measures on the test data and the OOB error indicate that the model is not over fitting.

The importance that the features of Table **??** had on each random forest model are given by Figures 19, 20, 21, 22 and 23. These show that the classifiers stochastic, make use of game and make use of length have no significant effect, and several of the features that are highlighted by the importance are inline with the correlation results. Moreover, the smoothing parameter k appears to no have a significant effect either. The most important features based on the random forest analysis were C_r/C_{median} and C_r/C_{mean}.

F Multivariate linear regression on median score

A multivariate linear regression has also been fitted to model the relationship between the features and the median score. The features included are given in Table 6 alongside their corresponding p values in distinct tournaments and their regression coefficients. Table 6 provides the results when considering tournaments with $p_n \leq 0.1$ and $p_e \leq 0.1$. The results when the probabilities are not constrained are given in Table 7.

G List of strategies

The strategies used in this study which are from Axelrod-Python library version 3.0.0.

Tournament type	Clustering Approach	Number of clusters	R^2 training data	R^2 test data	R^2 OOB score
standard	Approach 1	2	0.998831	0.987041	0.983708
	Approach 2	2	0.998643	0.978626	0.969202
	Approach 3	2	0.998417	0.985217	0.976538
	Approach 4	2	0.998794	0.990677	0.982959
noisy	Approach 1	2	0.997786	0.972229	0.968332
	Approach 2	2	0.997442	0.963254	0.955219
	Approach 3	2	0.997152	0.953164	0.940528
	Approach 4	3	0.996923	0.950728	0.935444
probabilistic ending	Approach 1	2	0.997909	0.981490	0.978120
	Approach 2	2	0.997883	0.973492	0.967150
	Approach 3	2	0.990448	0.890068	0.875822
	Approach 4	2	0.999636	0.995183	0.992809
noisy probabilistic ending	Approach 1	2	0.995347	0.957846	0.952353
	Approach 2	2	0.992813	0.909346	0.898613
	Approach 3	2	0.990579	0.824794	0.806540
	Approach 4	4	0.989465	0.841652	0.824052
over 45606 tournaments	Approach 1	2	0.997271	0.972914	0.969198
	Approach 2	2	0.996323	0.951194	0.940563
	Approach 3	2	0.993707	0.906941	0.891532
	Approach 4	3	0.993556	0.913335	0.898453

Table 5: Accuracy metrics for random forest models.

	Standard		Noisy		Probabilistic ending		Noisy probabilistic ending	
	R adjusted: 0.576		R adjusted: 0.561		R adjusted: 0.488		R adjusted: 0.762	
	Coefficient	p-value	Coefficient	p-value	Coefficient	p-value	Coefficient	p-value
constant	0.928	0.000	1.082	0.000	1.259	0.000	1.642	0.000
CC to C rate	0.043	0.000	0.104	0.000	0.024	0.000	-0.032	0.000
CD to C rate	-0.325	0.000	-0.052	0.000	-0.229	0.000	-0.110	0.000
DC to C rate	-0.204	0.000	-0.076	0.000	-0.102	0.000	-0.070	0.000
SSE	-0.294	0.000	-0.186	0.000	-0.131	0.000	-0.109	0.000
C_{max}	0.056	0.000	-0.060	0.011	-0.005	0.849	-0.240	0.000
C_{min}	0.156	0.000	-0.159	0.000	0.012	0.385	0.083	0.005
C_{mean}	1.838	0.000	2.247	0.000	1.822	0.000	2.030	0.000
C_{min} / C_r	-0.049	0.000	0.040	0.000	-0.018	0.000	-0.065	0.000
C_r / C_{mean}	0.552	0.000	-0.227	0.000	0.027	0.125	-0.043	0.000
k	-0.000	0.856	-0.000	0.245	-0.000	0.610	-0.000	0.572
n	-0.000	0.000	-	-	-	-	-	-
memory usage	0.010	0.000	0.004	0.000	-	-	-	-
C_r / C_{median}	-	-	0.252	0.000	0.181	0.000	-	-
p_n	-	-	0.770	0.000	-	-	0.494	0.000
p_e	-	-	-	-	0.763	0.000	0.285	0.000

Table 6: Results of multivariate linear regressions with the median score as the dependent variable. R squared is reported for each model. For noisy tournaments $p_n \leq 0.1$ and for probabilistic ending tournaments $p_e \leq 0.1$.

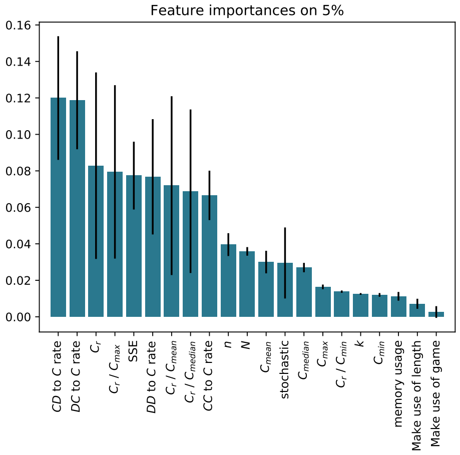

(a) Importance of features for clusters on 5% performance.

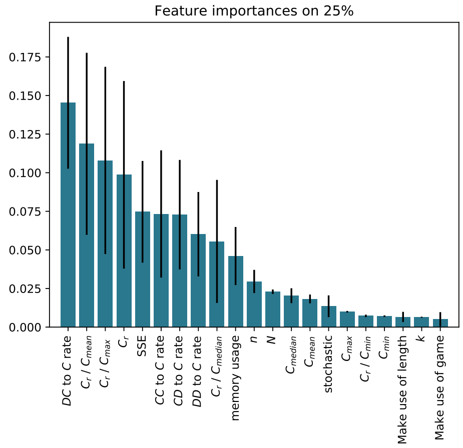

(b) Importance of features for clusters on 25% performance.

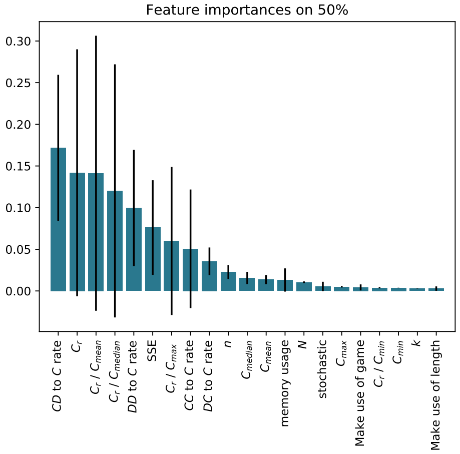

(c) Importance of features for clusters on 50% performance.

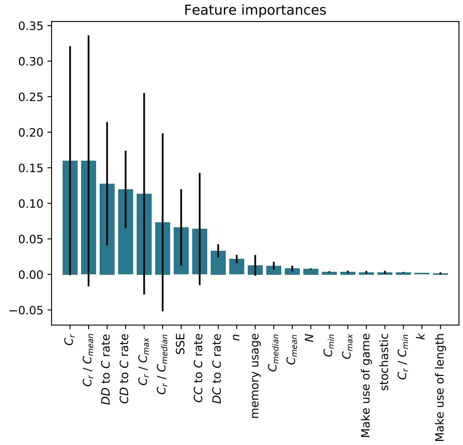

(d) Importance of features for clusters based on $k$means algorithm.

Figure 19: Importance of features in standard tournaments for different clustering methods.

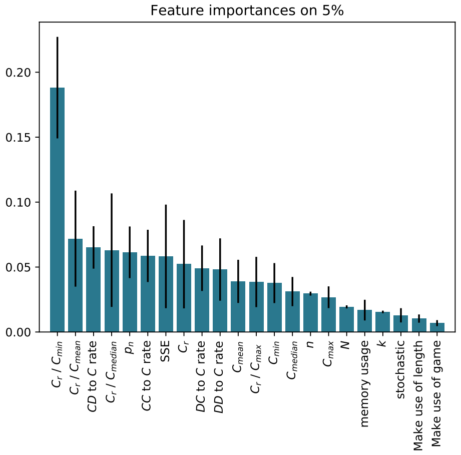

(a) Importance of features for clusters on 5% performance.

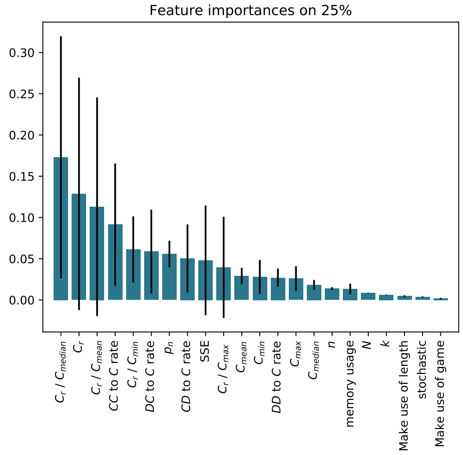

(b) Importance of features for clusters on 25% performance.

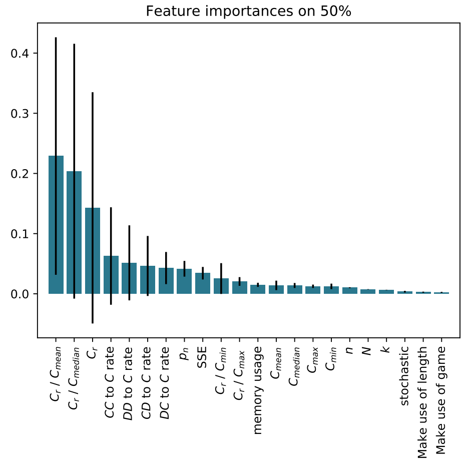

(c) Importance of features for clusters on 50% performance.

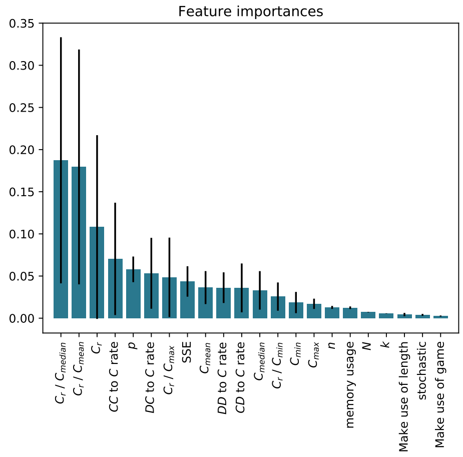

(d) Importance of features for clusters based on $k$means algorithm.

Figure 20: Importance of features in noisy tournaments for different clustering methods.

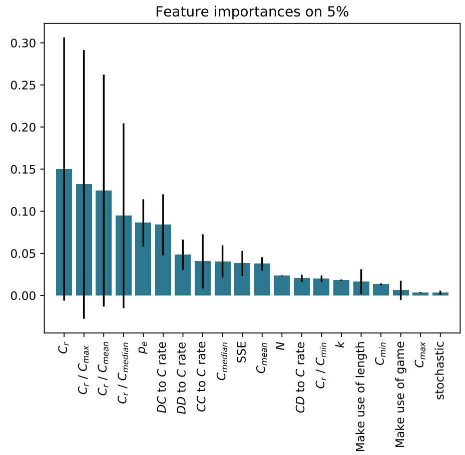

(a) Importance of features for clusters on 5% performance.

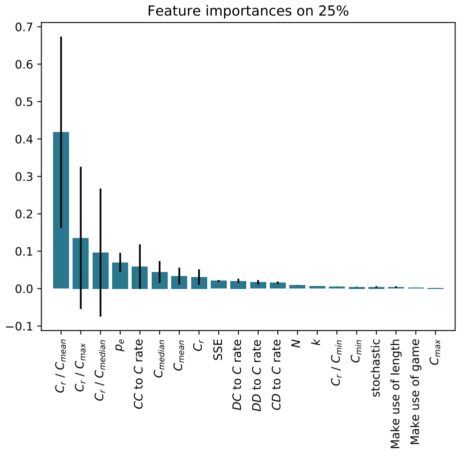

(b) Importance of features for clusters on 25% performance.

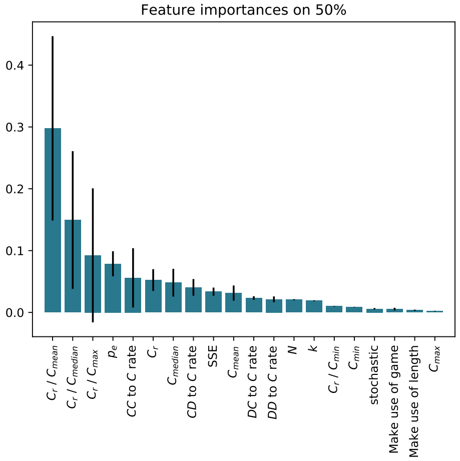

(c) Importance of features for clusters on 50% performance.

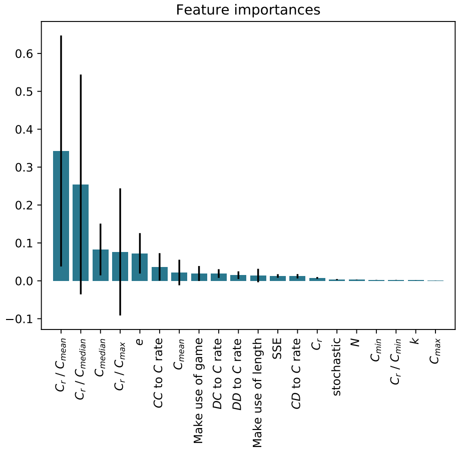

(d) Importance of features for clusters based on $k$means algorithm.

Figure 21: Importance of features in probabilistic ending tournaments for different clustering methods.

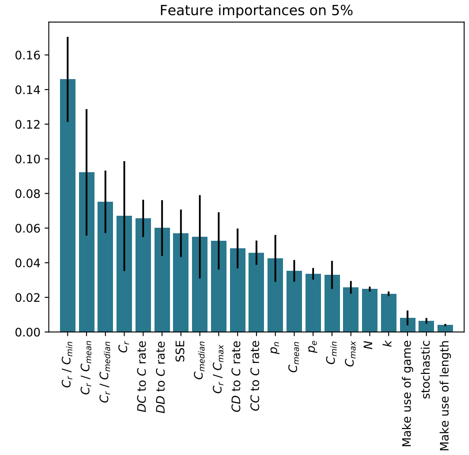

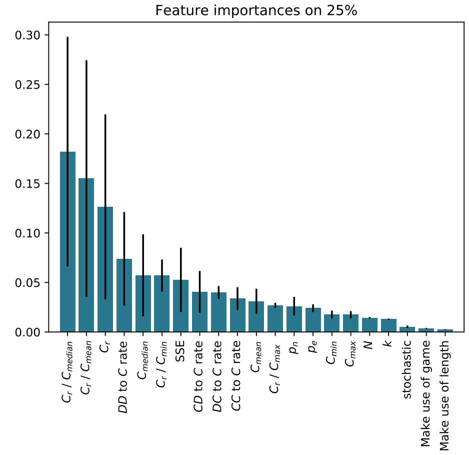

(a) Importance of features for clusters on 5% performance.

(b) Importance of features for clusters on 25% performance.

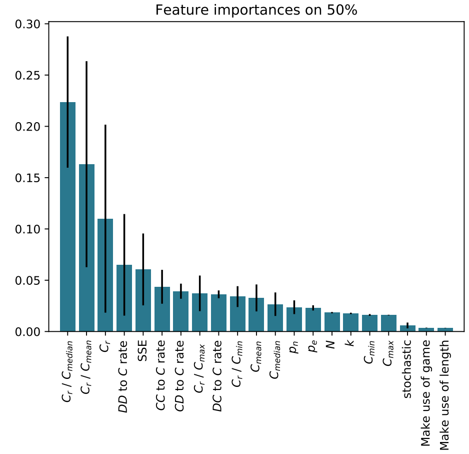

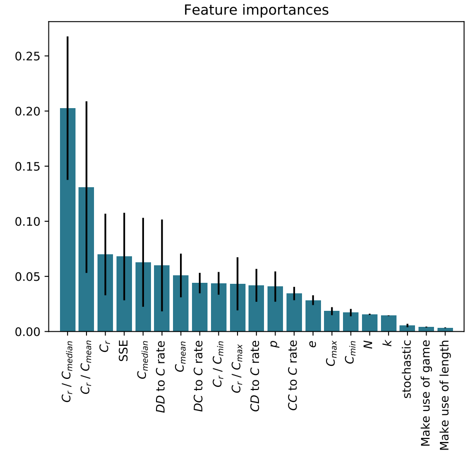

(c) Importance of features for clusters on 50% performance.

(d) Importance of features for clusters based on $k$means algorithm.

Figure 22: Importance of features in noisy probabilistic ending tournaments for different clustering methods.

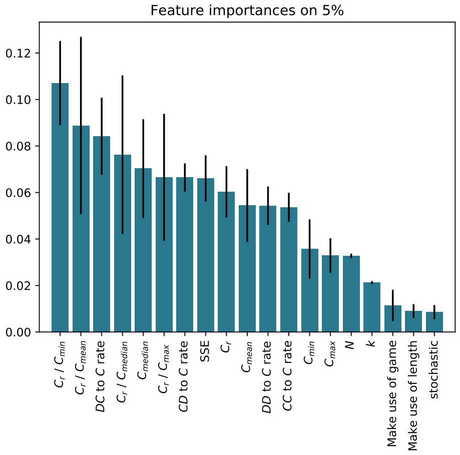

(a) Importance of features for clusters on 5% performance.

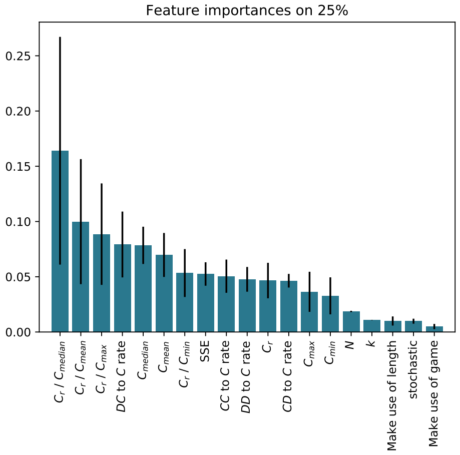

(b) Importance of features for clusters on 25% performance.

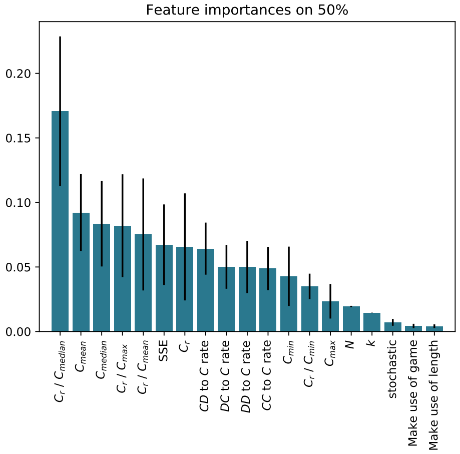

(c) Importance of features for clusters on 50% performance.

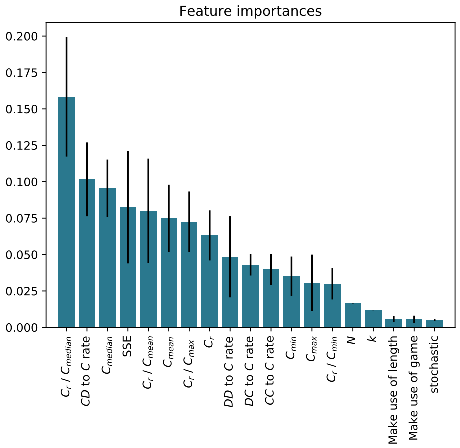

(d) Importance of features for clusters based on $k$means algorithm.

Figure 23: Importance of features over all the tournaments for different clustering methods.

	Standard		Noisy		Probabilistic ending		Noisy probabilistic ending	
	R adjusted: 0.575		R adjusted: 0.666		R adjusted: 0.816		R adjusted: 0.869	
	Coefficient	p-value	Coefficient	p-value	Coefficient	p-value	Coefficient	p-value
constant	0.928	0.000	2.143	0.000	2.466	0.000	1.824	0.000
CC to C rate	0.043	0.000	-0.468	0.000	0.223	0.000	-0.008	0.000
CD to C rate	-0.325	0.000	0.105	0.000	0.060	0.000	0.074	0.000
DC to C rate	-0.204	0.000	0.060	0.000	0.066	0.000	-0.002	0.011
SSE	-0.294	0.000	-0.365	0.000	0.055	0.000	-0.035	0.000
C_{max}	0.056	0.000	-	-	-0.045	0.081	-0.181	0.000
C_{min}	0.156	0.000	0.264	0.000	0.311	0.000	-	-
C_{mean}	1.838	0.000	2.046	0.000	1.506	0.000	2.273	0.000
C_{min} / C_r	-0.049	0.000	-0.252	0.000	-0.204	0.000	-	-
C_r / C_{mean}	0.552	0.000	-0.579	0.000	-1.137	0.000	-0.610	0.000
k	-0.000	0.856	0.000	0.788	0.000	0.009	0.000	0.005
n	-0.000	0.000	-	-	-	-	-	-
memory usage	0.010	0.000	-0.006	0.000	-	-	-	-
p_n	-	-	0.119	0.000	-	-	-	-
p_e	-	-	-	-	0.025	0.000	-0.028	0.000

Table 7: Results of multivariate linear regressions with the median score as the dependent variable. R squared is reported for each model.

1. ϕ [40]
2. π [40]
3. e [40]
4. ALLCorALLD [40]
5. Adaptive [28]
6. Adaptive Pavlov 2006 [25]
7. Adaptive Pavlov 2011 [28]
8. Adaptive Tit For Tat: 0.5 [41]
9. Aggravater [40]
10. Alexei [35]
11. Alternator [12, 32]
12. Alternator Hunter [40]
13. Anti Tit For Tat [24]
14. AntiCycler [40]
15. Appeaser [40]
16. Arrogant QLearner [40]
17. Average Copier [40]
18. Backstabber [40]
19. Better and Better [1]
20. Bully [33]
21. Calculator [1]
22. Cautious QLearner [40]
23. Champion [11]
24. CollectiveStrategy [29]
25. Contrite Tit For Tat [43]
26. Cooperator [12, 32, 36]
27. Cooperator Hunter [40]
28. Cycle Hunter [40]
29. Cycler CCCCCD [40]
30. Cycler CCCD [40]
31. Cycler CCCDCD [40]
32. Cycler CCD [32]
33. Cycler DC [40]
34. Cycler DDC [32]
35. DBS [9]
36. Davis [10]
37. Defector [12, 32, 36]
38. Defector Hunter [40]
39. Double Crosser [40]
40. Desperate [42]
41. DoubleResurrection [18]
42. Doubler [1]
43. Dynamic Two Tits For Tat [40]
44. EasyGo [28, 1]
45. Eatherley [11]
46. Eventual Cycle Hunter [40]
47. Evolved ANN [40]
48. Evolved ANN 5 [40]
49. Evolved ANN 5 Noise 05 [40]
50. Evolved FSM 16 [40]
51. Evolved FSM 16 Noise 05 [40]
52. Evolved FSM 4 [40]
53. Evolved HMM 5 [40]
54. EvolvedLookerUp1 1 1 [40]
55. EvolvedLookerUp2 2 2 [40]

56. Eugine Nier [35]
57. Feld [10]
58. Firm But Fair [19]
59. Fool Me Forever [40]
60. Fool Me Once [40]
61. Forgetful Fool Me Once [40]
62. Forgetful Grudger [40]
63. Forgiver [40]
64. Forgiving Tit For Tat [40]
65. Fortress3 [7]
66. Fortress4 [7]
67. GTFT [21, 34]
68. General Soft Grudger [40]
69. Gradual [14]
70. Gradual Killer [1]
71. Grofman[10]
72. Grudger [10, 13, 14, 42, 28]
73. GrudgerAlternator [1]
74. Grumpy [40]
75. Handshake [37]
76. Hard Go By Majority [32]
77. Hard Go By Majority: 10 [40]
78. Hard Go By Majority: 20 [40]
79. Hard Go By Majority: 40 [40]
80. Hard Go By Majority: 5 [40]
81. Hard Prober [1]
82. Hard Tit For 2 Tats [39]
83. Hard Tit For Tat [2]
84. Hesitant QLearner[40]
85. Hopeless [42]
86. Inverse [40]
87. Inverse Punisher [40]
88. Joss [10, 39]
89. Knowledgeable Worse and Worse [40]
90. Level Punisher [18]
91. Limited Retaliate 2 [40]
92. Limited Retaliate 3 [40]
93. Limited Retaliate [40]
94. MEM2 [30]
95. Math Constant Hunter [40]
96. Meta Hunter Aggressive [40]
97. Meta Hunter [40]
98. Meta Majority [40]
99. Meta Majority Finite Memory [40]
100. Meta Majority Long Memory [40]
101. Meta Majority Memory One [40]
102. Meta Minority [40]
103. Meta Mixer [40]
104. Meta Winner [40]
105. Meta Winner Deterministic [40]
106. Meta Winner Ensemble [40]
107. Meta Winner Finite Memory [40]
108. Meta Winner Long Memory [40]
109. Meta Winner Memory One [40]
110. Meta Winner Stochastic [40]
111. NMWE Deterministic [40]
112. NMWE Finite Memory [40]
113. NMWE Long Memory [40]
114. NMWE Memory One [40]
115. NMWE Stochastic [40]
116. Naive Prober [28]
117. Negation [2]
118. Nice Average Copier [40]
119. Nice Meta Winner [40]
120. Nice Meta Winner Ensemble [40]
121. Nydegger [10]
122. Omega TFT [25]
123. Once Bitten [40]
124. Opposite Grudger [40]
125. PSO Gambler 1 1 1 [40]
126. PSO Gambler 2 2 2 [40]
127. PSO Gambler 2 2 2 Noise 05 [40]
128. PSO Gambler Mem1 [40]
129. Predator [7]
130. Prober [28]
131. Prober 2 [1]
132. Prober 3 [1]
133. Prober 4 [1]
134. Pun1 [7]
135. Punisher [40]
136. Raider [8]
137. Random Hunter [40]
138. Random: 0.5 [10, 41]
139. Remorseful Prober [28]
140. Resurrection [18]
141. Retaliate 2 [40]
142. Retaliate 3 [40]
143. Retaliate [40]
144. Revised Downing [10]

145. Ripoff [6]
146. Risky QLearner [40]
147. SelfSteem [17]
148. ShortMem [17]
149. Shubik [10]
150. Slow Tit For Two Tats [40]
151. Slow Tit For Two Tats 2 [1]
152. Sneaky Tit For Tat [40]
153. Soft Go By Majority [12, 32]
154. Soft Go By Majority 10 [40]
155. Soft Go By Majority 20 [40]
156. Soft Go By Majority 40 [40]
157. Soft Go By Majority 5 [40]
158. Soft Grudger [28]
159. Soft Joss [1]
160. SolutionB1 [5]
161. SolutionB5 [5]
162. Spiteful Tit For Tat [1]
163. Stalker [16]
164. Stein and Rapoport [10]
165. Stochastic Cooperator [3]
166. Stochastic WSLS [40]
167. Suspicious Tit For Tat [14, 24]
168. TF1 [40]
169. TF2 [40]
170. TF3 [40]
171. Tester [11]
172. ThueMorse [40]
173. ThueMorseInverse [40]
174. Thumper [6]
175. Tit For 2 Tats (**Tf2T**) [12]
176. Tit For Tat (**TFT**) [10]
177. Tricky Cooperator [40]
178. Tricky Defector [40]
179. Tullock [10]
180. Two Tits For Tat (**2TFT**) [12]
181. VeryBad [17]
182. Willing [42]
183. Win-Shift Lose-Stay (**WShLSt**) [28]
184. Win-Stay Lose-Shift (**WSLS**) [26, 34, 39]
185. Winner12 [31]
186. Winner21 [31]
187. Worse and Worse[1]
188. Worse and Worse 2[1]
189. Worse and Worse 3[1]
190. ZD-Extort-2 v2 [27]
191. ZD-Extort-2 [39]
192. ZD-Extort-4 [40]
193. ZD-GEN-2 [27]
194. ZD-GTFT-2 [39]
195. ZD-SET-2 [27]

References

[1] Lifl (1998) prison. `http://www.lifl.fr/IPD/ipd.frame.html`. Accessed: 2017-10-23.

[2] The prisoner's dilemma. http://www.prisoners-dilemma.com/, 2017.

[3] C. Adami and A. Hintze. Evolutionary instability of zero-determinant strategies demonstrates that winning is not everything. *Nature communications*, 4(1):2193, 2013.

[4] D. Arthur and S. Vassilvitskii. k-means++: The advantages of careful seeding. In *Proceedings of the eighteenth annual ACM-SIAM symposium on Discrete algorithms*, pages 1027–1035. Society for Industrial and Applied Mathematics, 2007.

[5] D. Ashlock, J. A. Brown, and P. Hingston. Multiple opponent optimization of prisoner's dilemma playing agents. *IEEE Transactions on Computational Intelligence and AI in Games*, 7(1):53–65, 2015.

[6] D. Ashlock and E. Y. Kim. Fingerprinting: Visualization and automatic analysis of prisoner's dilemma strategies. *IEEE Transactions on Evolutionary Computation*, 12(5):647–659, 2008.

[7] W. Ashlock and D. Ashlock. Changes in prisoner's dilemma strategies over evolutionary time with different population sizes. In *2006 IEEE International Conference on Evolutionary Computation*, pages 297–304. IEEE, 2006.

[8] W. Ashlock, J. Tsang, and D. Ashlock. The evolution of exploitation. In *2014 IEEE Symposium on Foundations of Computational Intelligence (FOCI)*, pages 135–142. IEEE, 2014.

[9] T. C. Au and D. Nau. Accident or intention: that is the question (in the noisy iterated prisoner's dilemma). In *Proceedings of the fifth international joint conference on Autonomous agents and multiagent systems*, pages 561–568. ACM, 2006.

[10] R. Axelrod. Effective choice in the prisoner's dilemma. *Journal of Conflict Resolution*, 24(1):3–25, 1980.

[11] R. Axelrod. More effective choice in the prisoner's dilemma. *Journal of Conflict Resolution*, 24(3):379–403, 1980.

[12] R. Axelrod and W. D. Hamilton. The evolution of cooperation. *science*, 211(4489):1390–1396, 1981.

[13] J. S. Banks and R. K. Sundaram. Repeated games, finite automata, and complexity. *Games and Economic Behavior*, 2(2):97–117, 1990.

[14] B. Beaufils, J. P. Delahaye, and P. Mathieu. Our meeting with gradual, a good strategy for the iterated prisoner's dilemma. In *Proceedings of the Fifth International Workshop on the Synthesis and Simulation of Living Systems*, pages 202–209, 1997.

[15] L. Breiman. Random forests. *Machine learning*, 45(1):5–32, 2001.

[16] A. Carvalho, H. Rocha, F. Amaral, and F. Guimaraes. Iterated prisoner's dilemma-an extended analysis. *Iterated Prisoner's Dilemma-An extended analysis*, 2013.

[17] A. Carvalho, H. P. Rocha, F. T. Amaral, and F. G. Guimaraes. Iterated prisoner's dilemma-an extended analysis. 2013.

[18] Arnold Eckhart. Coopsim v0.9.9 beta 6. `https://github.com/jecki/CoopSim/`, 2015.

[19] M. R. Frean. The prisoner's dilemma without synchrony. *Proceedings of the Royal Society of London B: Biological Sciences*, 257(1348):75–79, 1994.

[20] D. Fudenberg and E. Maskin. The folk theorem in repeated games with discounting or with incomplete information. In *A Long-Run Collaboration On Long-Run Games*, pages 209–230. World Scientific, 2009.

[21] M. Gaudesi, E. Piccolo, G. Squillero, and A. Tonda. Exploiting evolutionary modeling to prevail in iterated prisoner's dilemma tournaments. *IEEE Transactions on Computational Intelligence and AI in Games*, 8(3):288–300, 2016.

[22] M. Harper, V. Knight, M. Jones, G. Koutsovoulos, N. E. Glynatsi, and O. Campbell. Reinforcement learning produces dominant strategies for the iterated prisoner's dilemma. *PloS one*, 12(12):e0188046, 2017.

[23] T. Hastie, R. Tibshirani, J. Friedman, and J. Franklin. The elements of statistical learning: data mining, inference and prediction. *The Mathematical Intelligencer*, 27(2):83–85, 2005.

[24] C. Hilbe, M. A. Nowak, and A. Traulsen. Adaptive dynamics of extortion and compliance. *PloS one*, 8(11):e77886, 2013.

[25] G. Kendall, X. Yao, and S. Y. Chong. *The iterated prisoners' dilemma: 20 years on*, volume 4. World Scientific, 2007.

[26] D. Kraines and V. Kraines. Pavlov and the prisoner's dilemma. *Theory and decision*, 26(1):47–79, 1989.

[27] S. Kuhn. Prisoner's dilemma. In Edward N. Zalta, editor, *The Stanford Encyclopedia of Philosophy*. Metaphysics Research Lab, Stanford University, spring 2017 edition, 2017.

[28] J. Li, P. Hingston, S. Member, and G. Kendall. Engineering Design of Strategies for Winning Iterated Prisoner ' s Dilemma Competitions. 3(4):348–360, 2011.

[29] J. Li and G. Kendall. A strategy with novel evolutionary features for the iterated prisoner's dilemma. *Evolutionary Computation*, 17(2):257–274, 2009.

[30] J. Li, G. Kendall, and S. Member. The effect of memory size on the evolutionary stability of strategies in iterated prisoner ' s dilemma. X(X):1–8, 2014.

[31] P. Mathieu and J. P. Delahaye. New winning strategies for the iterated prisoner's dilemma. *Journal of Artificial Societies and Social Simulation*, 20(4):12, 2017.

[32] S. Mittal and K. Deb. Optimal strategies of the iterated prisoner's dilemma problem for multiple conflicting objectives. *IEEE Transactions on Evolutionary Computation*, 13(3):554–565, 2009.

[33] J. H. Nachbar. Evolution in the finitely repeated prisoner's dilemma. *Journal of Economic Behavior & Organization*, 19(3):307–326, 1992.

[34] M. Nowak and K. Sigmund. A strategy of win-stay, lose-shift that outperforms tit-for-tat in the prisoner's dilemma game. *Nature*, 364(6432):56, 1993.

[35] prase. Prisoner's dilemma tournament results. `https://www.lesswrong.com/posts/hamma4XgeNrsvAJv5/prisoner-s-dilemma-tournament-results`, 2011.

[36] W. H. Press and F. J. Dyson. Iterated prisoner's dilemma contains strategies that dominate any evolutionary opponent. *Proceedings of the National Academy of Sciences*, 109(26):10409–10413, 2012.

[37] A. J. Robson. Efficiency in evolutionary games: Darwin, nash and the secret handshake. *Journal of theoretical Biology*, 144(3):379–396, 1990.

[38] P. J. Rousseeuw. Silhouettes: a graphical aid to the interpretation and validation of cluster analysis. *Journal of computational and applied mathematics*, 20:53–65, 1987.

[39] A. J. Stewart and J. B. Plotkin. Extortion and cooperation in the prisoner's dilemma. *Proceedings of the National Academy of Sciences*, 109(26):10134–10135, 2012.

[40] The Axelrod project developers. Axelrod: 3.0.0. `http://dx.doi.org/10.5281/zenodo.807699`, April 2016.

[41] E. Tzafestas. Toward adaptive cooperative behavior. *From Animals to animals: Proceedings of the 6th International Conference on the Simulation of Adaptive Behavior (SAB-2000)*, 2:334–340, 2000.

[42] P. Van den Berg and F. J. Weissing. The importance of mechanisms for the evolution of cooperation. *Proceedings of the Royal Society B: Biological Sciences*, 282(1813):20151382, 2015.

[43] J. Wu and R. Axelrod. How to cope with noise in the iterated prisoner's dilemma. *Journal of Conflict resolution*, 39(1):183–189, 1995.
