# Supplementary material for: Properties of winning Iterated Prisoner’s Dilemma strategies
Source: PLoS Comput Biol. 2024 Dec 26;20(12):e1012644. doi: 10.1371/journal.pcbi.1012644 (PMC11670945; doi:10.1371/journal.pcbi.1012644)
Supplement: Attachment — Submitted filename: Point-by-point.pdf [file pcbi.1012644.s002.pdf]

First, we would like to thank the editor and reviewers for their valuable feedback. Their comments have been helpful and constructive, and we have revised our manuscript accordingly. In particular, we have made the following changes:

- Motivated by a comment from Reviewer #1, we have expanded the discussion to include further work and revised the introduction to address why such a study was not feasible until now.

- Reviewer #1 suggested adding more detail about the Axelrod-Python packages. We have included additional explanations to clarify this aspect of our work.

- As suggested by Reviewer #1, we have added references to relevant statements throughout the manuscript.

- In line with Reviewer #1's suggestion, we have enriched the captions to provide more details for each figure.

- Reviewer #2's comments, we have clarified the measures in the main text, which were previously only explained in the figure captions.

Please find a detailed response to their comments below.

Editor Remarks to Author:

Thank you very much for submitting your manuscript "Properties of Winning Iterated Prisoner's Dilemma Strategies" for consideration at PLOS Computational Biology. As with all papers reviewed by the journal, your manuscript was reviewed by members of the editorial board and by several independent reviewers. The reviewers appreciated the attention to an important topic. Based on the reviews, we are likely to accept this manuscript for publication, providing that you modify the manuscript according to the review recommendations.

Reply: Thank you for considering our paper and for providing positive feedback. The reviewers' comments have been very helpful. Please find all details about how we addressed each comment raised by the reiweres in our response to the reviewers below.

Reviewer #1:

Comment: Summary: This paper explores the performance of strategies in the IPD. This research analyses 195 strategies across thousands of computer tournaments, examining what makes certain strategies more successful in diverse IPD environments. The study used four different types of tournaments: Standard tournaments, Noisy tournaments, Probabilistic ending tournaments and Noisy probabilistic ending tournaments. They find that no single strategy excels in all settings. However, several key traits appear in successful strategies, particularly the ability to adjust to the environment and population dynamics.

Thanks for this submission, I find the text easy to read and all the concepts well explained.

Reply: Thank you for carefully reading our manuscript, and for the constructive feedback below.

Comment: This seems like a low-hanging fruit idea for someone like Axelrod-Python. If they have all of these strategies, what is the best, after all, they maintain and add new strategies to the library, why do you think this hasn't been done before? I feel the data analysis and the interpretation is sound, but maybe can you add other efforts to do the same, and what do you do differently?

Reply: We agree with the reviewer's comment that this could be considered a ``low-hanging fruit'' idea for the developers of the Axelrod-Python library. We understand that the reviewer is asking whether this type of analysis has been done before; if so, what did we do differently?

To start, we would like to emphasize that this analysis would not have been feasible before the development of the Axelrod-Python library. Previously, researchers would have needed to locate, understand, and implement code for each strategy from the literature in order to conduct a large-scale tournament. Additionally, they would have had to develop all the necessary code to manage the tournament, a challenging task for any researcher. The creation of the Axelrod-Python package has made such an analysis feasible. This package is the result of a collaborative effort that has produced a substantial database of strategies from the literature. We would also like to highlight that the library's open-source nature has encouraged contributions from a broad user base.

Since the package's development, we believe the reasons for limited comprehensive analyses are rooted in field conventions. In our field, tournaments or evolutionary dynamics often rely on a select list of hand picked strategies chosen by modelers, typically based on specific properties they wish to examine. This practice may stem from certain misconceptions, such as the assumption that because Tit-for-Tat performs relatively well, testing against only Tit-for-Tat variants is sufficient. Another misconception may arise from the Press & Dyson result, which suggests that lower memory strategies will always dominate in pairwise interactions, leading some researchers to consider only memory-one strategies. However, this result strictly applies only to pairwise interactions.

We did find one example where the setup of the tournament itself was questioned. Specifically, in Is Tit-for-Tat the Answer? On the Conclusions drawn from Axelrod's Tournaments (Rapoport, 2015), Rapoport examined the effects of variations in format, objective criteria, and payoff values on tournament outcomes. However, their study analyzed different effects than ours and used a single tournament setup with a specific selection of strategies. They focused on Axelrod's original tournament and demonstrated how certain changes affected the outcome. For example, including strategies like the "kingmakers" positively impacted the performance of Tit For Tat.

Changes: We have updated the introduction to clearly state why such an analysis has not been done before, and we discuss the work of Rapoport (2015).

Comment: You mention somewhere that there are evolutionary approaches to this, specifically evolutionary game theory approaches. Maybe adding why you chose to do computer simulations instead? (I know that EGT has some constraints, but highlight why you do how you do it to overcome those constraints).

Reply: Excellent observation. Many strategies, such as Win-Stay-Lose-Shift and Generous Tit For Tat, emerged due to their strong performance in evolutionary dynamics. Axelrod's original work involved computer tournaments, so we chose to remain consistent with this approach since this comprehensive study had not yet been undertaken.

Moreover, in evolutionary dynamics, there are several factors to consider, such as which evolutionary process to apply and the high computational cost. However, it remains an interesting question. For example, we could consider strategy performance in terms of fixation probabilities in the case of low mutation rates. This is future work we would be eager to pursue.

Changes: We've added a paragraph to the discussion section outlining potential extensions into evolutionary dynamics.

Comment: I don't know if it's enough to say that you used Axelrod-Python, but maybe also add how these tournaments are performed? I haven't used the tool, so I don't know how they simulate those exactly. I feel the library itself is central to how the results are generated, so maybe expand a bit on that.

Reply: Thank you for this suggestion. We agree that many readers may not be familiar with the Axelrod-Python package.

Changes: We've expanded the methodology section to include more details on how to run tournaments using the Axelrod library. We have included a figure showing a code example of how to run such a tournament.

Comment: Check for references when you make statements.

Reply: We thank the reviewer for this comment.

Changes: We have added references to the sentences indicated by the reviewer and in addition the following sentences:

- "Specifically, much like the recognized value of diversity in training datasets, such as variations in image perspective,..."

- "Already well-known in the AI/ML literature, adding noise to training data leads to more robust models"

Comment: I believe it's a matter of style, but I prefer if figures are self-explanatory. As you did with Figure 1, where you guide the reader through what is shown, Table 4 and Figures 3,4 and 5 lack some context in the figure labels, especially in figures like 5, some guidance is appreciated.

Reply: We agree that figures should be self-explanatory.

Changes: We have included additional details in the figures and tables that were previously missing. These were Table 4 and Figures 3, 4, and 5 (now Figures 4, 5, and 6).

Reviewer #2:

Comment: The authors uncover properties of winning strategies in the iterated Prisoner's Dilemma (IPD), one of the most renowned paradigms for studying cooperation over the past decades. They consider a large collection of 195 strategies in thousands of computer tournaments and conduct a thorough analysis of their performance. Their conclusions refine the properties described by Axelrod: a successful strategy needs to be nice, provocable, generous, somewhat envious, clever, and adaptable to the environment.

There are several aspects of this work that I appreciate greatly. First, the inclusiveness of the study design is commendable: it not only includes all kinds of strategies from IPD literature but also incorporates variations in the tournaments (such as random noise and match length). Second, their findings challenge some of Axelrod's suggestions, particularly the advice to "not be clever" and "not be envious." Additionally, I find the property of being adaptable to the environment—explained by the authors as a strategy's cooperation rate compared to the average cooperation rate in a tournament—very inspiring.

Reply: Thank you for your comprehensive summary of our work! We appreciate the comments.

Comment: On page 8, I suggest including the explanations for C_max, C_median, C_mean, and SSE error in the main text, as they currently seem to be defined only in figures and tables.

Reply: Thank you for this suggestion. These measures should indeed be explained in the main text as well.

Changes: We have included explanations of each measure in the main text, in section "Model".

Comment: In Table 3 on page 9, there is a contradiction between "A strategy's cooperating rate divided by the minimum" and "C_min/C_r."

Reply: Correct. That is a typo. We have addressed this.

Comment: On page 13, the sentence "envious strategies capable of both exploiting and not their opponents…" appears to be incomplete.

Reply: This is indeed correct; the sentence is incomplete. The sentence should read: ``We also showed that while the type of exploitation attempted by ZDs is not typically effective in standard tournaments, envious strategies capable of both exploiting and not their opponents can be highly successful''.
